# Pan-cancer single cell and spatial transcriptomics analysis deciphers the molecular landscapes of senescence related cancer-associated fibroblasts and reveals its predictive value in neuroblastoma via integrated multi-omics analysis and machine learning

**DOI:** 10.3389/fimmu.2024.1506256

**Published:** 2024-12-05

**Authors:** Shan Li, Junyi Luo, Junhong Liu, Dawei He

**Affiliations:** ^1^ Department of Urology, Children’s Hospital of Chongqing Medical University, Chongqing, China; ^2^ Chongqing Key Laboratory of Children Urogenital Development and Tissue Engineering, Children’s Hospital of Chongqing Medical University, Chongqing, China; ^3^ China International Science and Technology Cooperation base of Child Development and Critical Disorders, National Clinical Research Center for Child Health and Disorders, Ministry of Education Key Laboratory of Child Development and Disorders, Chongqing Key Laboratory of Pediatrics, Children’s Hospital of Chongqing Medical University, Chongqing, China; ^4^ Department of Day Surgery, National Clinical Research Center for Child Health and Disorders, Ministry of Education, Key Laboratory of Child Development and Disorder, Children’s Hospital of Chongqing Medical University, Chongqing, China

**Keywords:** pan-cancer analysis, neuroblastoma, cancer-associated fibroblasts, senescence, multi-omics analysis, machine learning, prognostic prediction

## Abstract

**Introduction:**

Cancer-associated fibroblasts (CAFs) are a diverse group of cells that significantly contribute to reshaping the tumor microenvironment (TME), and no research has systematically explored the molecular landscapes of senescence related CAFs (senes CAF) in NB.

**Methods:**

We utilized pan-cancer single cell and spatial transcriptomics analysis to identify the subpopulation of senes CAFs via senescence related genes, exploring its spatial distribution characteristics. Harnessing the maker genes with prognostic significance, we delineated the molecular landscapes of senes CAFs in bulk-seq data. We established the senes CAFs related signature (SCRS) by amalgamating 12 and 10 distinct machine learning (ML) algorithms to precisely diagnose stage 4 NB and to predict prognosis in NB. Based on risk scores calculated by prognostic SCRS, patients were categorized into high and low risk groups according to median risk score. We conducted comprehensive analysis between two risk groups, in terms of clinical applications, immune microenvironment, somatic mutations, immunotherapy, chemotherapy and single cell level. Ultimately, we explore the biological function of the hub gene JAK1 in pan-cancer multi-omics landscape.

**Results:**

Through integrated analysis of pan-cancer spatial and single-cell transcriptomics data, we identified distinct functional subgroups of CAFs and characterized their spatial distribution patterns. With marker genes of senes CAF and leave-one-out cross-validation, we selected RF algorithm to establish diagnostic SCRS, and SuperPC algorithm to develop prognostic SCRS. SCRS demonstrated a stable predictive capability, outperforming the previously published NB signatures and clinic variables. We stratified NB patients into high and low risk group, which showed the low-risk group with a superior survival outcome, an abundant immune infiltration, a different mutation landscape, and an enhanced sensitivity to immunotherapy. Single cell analysis reveals biologically cellular variations underlying model genes of SCRS. Spatial transcriptomics delineated the molecular variant expressions of hub gene JAK1 in malignant cells across cancers, while immunohistochemistry validated the differential protein levels of JAK1 in NB.

**Conclusion:**

Based on multi-omics analysis and ML algorithms, we successfully developed the SCRS to enable accurate diagnosis and prognostic stratification in NB, which shed light on molecular landscapes of senes CAF and clinical utilization of SCRS.

## Introduction

Neuroblastoma (NB), a common extracranial solid tumor in children, presents both challenges and opportunities for innovative diagnostics and treatment plans (1). While NB accounts for only 6-10% of pediatric tumors, it is responsible for up to 12-15% of cancer-related deaths in children (2). The prognosis for NB patients varies significantly: patients with low to intermediate-risk disease experience a 5-year event-free survival (EFS) exceeding 80%, whereas the high-risk group, comprising about half of all cases, faces a 5-year EFS of merely 50% (3). Researchers often employ the International Neuroblastoma Staging System (INSS) for diagnosis. Patients over one year of age with metastatic disease are typically classified as INSS Stage 4 NB, with a 5-year EFS of approximately 50% (4).

NB growths exhibit remarkable diversity and heterogeneity, with neoplastic cells engaging in complex dialogues with their surrounding microenvironment, creating an intricate biological system (5). Among the various cellular populations within the tumor milieu, cancer-associated fibroblasts (CAFs) have emerged as a dominant and numerous group (6), attracting considerable scientific interest recently. The nuanced interplay between CAFs, stromal elements, and immune components critically shapes the restructuring of the tumor microenvironment. This process encompasses the formation of new blood vessels, alterations to the extracellular scaffold, and mechanisms for evading immune detection (7). Notably, most current therapeutic approaches, including those targeting the immune system and cytotoxic agents, have largely neglected the pivotal role of CAFs. Our grasp of how CAFs interact with other tumor milieu constituents remains inadequate for developing robust treatment protocols. Additional investigation is essential to enhance our comprehension of these relationships and lay the groundwork for potent clinical interventions.

Cellular senescence, a distinctive state acquired by stromal elements like CAFs, confers unique immunomodulatory capabilities (8). This condition can be induced by diverse stressors, including oncogene activation, DNA-damaging agents, and oxidative imbalance, all converging on persistent genomic injury signaling (9). The subsequent activation of p53/p21 and p16/Rb tumor suppressor cascades leads to an irreversible halt in cell division, functioning as a potent intrinsic defense against malignant transformation. The extrinsic effects of senescence are mediated by senescence-associated secretory phenotypes (SASP), encompassing context-specific extracellular matrix components, growth modulators, cytokines, and immunological messengers (10). Senescent stromal cells have been proved to play a role in SASP-mediated alterations of the milieu in chronic inflammatory and fibrotic conditions, cancer included. In liver cancer models, for instance, senescence of activated hepatic stellate cells limited scarring by reducing matrix deposition and enhances immune-mediated clearance through increased IL6 and IFNγ production, promoting natural killer cell and macrophage activity (11). Contrastingly, in squamous carcinoma studies, IL6 secretion of senescent fibroblasts leads to granulocyte infiltration and impaired CD8+ T-cell function (12). Thus, SASP composition varies greatly depending on context, with senescence capable of exerting both tumor-promoting and tumor-suppressing influences within the tumor microenvironment (8). However, the therapeutic mechanisms of senescence related CAFs (senes CAFs) in NB remain poorly understood, with limited research on senes CAFs related genes and their prognostic and diagnostic value for NB patients.

The landscape of medical research is experiencing a profound shift, propelled by cutting-edge bioinformatics tools. Novel approaches in gene expression analysis, genetic variation mapping, and high-resolution single cell studies are reshaping our understanding of disease mechanisms. These methodologies, particularly when applied to the study of senes CAFs in NB, offer unprecedented insights into potential therapeutic avenues. Leveraging multiple machine learning algorithms (13), our goal was to establish a novel model based on senes CAFs related genes for identifying INSS 4 NB patients, evaluating the efficacy of immunotherapy, and predicting patient outcomes, by utilizing extensive multi-omics sequencing data.

## Materials and methods

### Source data

Analyzing the bulk-seq data, we obtained five transcriptome datasets of NB: GSE49710 and GSE85047 sourced from GEO database, TARGET-NB from TARGET database, and E-MTAB-8248 and E-MTAB-179 from ArrayExpress database. We utilized the log2 (x+1) algorithm to normalize transcriptomic data, and conducted the combat function of the “sva” R package to solve batch effects (14). After excluding patients with incomplete follow-up data, we incorporated 1617 patients in total. We used the GSE49710 cohort as the training cohort to develop both prognostic and diagnostic signatures, contrastingly, the remaining four cohorts served as validation cohorts. Comprehensive patient information in bulk-seq cohorts was listed in **Supplementary Table S1**. Moreover, scRNA-seq datasets of NB (GSE137804, GSE192906 and GSE140819) were acquired from the GEO database, with detailed clinic information of NB patients in GSE137804 included in **Supplementary Table S2**. Another NB scRNA-seq cohort was download from https://www.neuroblastomacellatlas.org/. There is no requirement for ethical approval or patient consent, as the bioinformatics data in our analysis is publicly available. The general workflow of our study was illustrated in **Supplementary Figure S1A**. For pan-cancer single cell data, GSE176078, GSE203612, GSE138709, GSE142784, GSE166555, GSE181919, GSE149614, GSE131907, GSE184880, GSE215120 and GSE139829 datasets were sourced from the GEO database for analysis. The scRNA-seq data of PRAD from Chen et al. were downloaded from http://www.pradcellatlas.com/. Genes related to senescence were sourced from Molecular Signatures Database (MSigDB) (https://www.gsea-msigdb.org/gsea/msigdb/cards/FRIDMAN_SENESCENCE_UP.html). This search identified 77 senescence related genes, detailed in **Supplementary Table S3**.

### Single cell analysis of senescence related CAFs

For pan-cancer scRNA-seq data, raw gene expression matrices were loaded into a Seurat object and read into R software via Seurat R package (15). We removed low-quality cells according to criteria of >40,000 UMI per cell, <500 genes per cell, >5,000 genes per cell and >20% mitochondrial genes. For scRNA-seq data retrieved from inDrop platform, we filtered out cells with UMI counts >40,000, gene counts <200, gene counts >5000, and mitochondrial gene count >20%. Doublets were excluded by DoubletFinder R package (16). The “Harmony” R package was used to remove technical batch effects while keeping biological variance after batch consolidation (17). The local inverse Simpson’s Index (LISI) was utilized to evaluate corrections of batch effects (17). For analysis of NB, scRNA-seq data of GSE137804 was loaded as Seurat object via “Seurat” R package (15). We performed quality control to delete low-quality cells with feature counts above 7500 or below 300, or mitochondrial counts above 20%. The function FindVariableFeatures was used to identify the top 2000 genes with most cell variants (18), at which PCA was conducted. The FindClusters function was employed to identify different clusters at a resolution of 0.5. We performed the RunUMAP function to reduce dimensions and manually annotated major cell types based on canonical markers (5). With the makers of CAF subtypes previously reported (19, 20), various clusters of CAFs were identified. Obtaining markers of CAF subtypes was performed with the criteria of log2FC >0.25 and p value <0.05 by the FindAllMarkers function, illustrated in **Supplementary Table S4**. Seven single cell scoring algorithms (AUCell in “AUCell” R package, Ucell in “Ucell” R package, ssGSEA and GSVA in “GSVA” R package, singscore in “singscore” R package, AddModuleScore and PercentageFeatureSet in “Seurat” R package) were applied to perform enrichment scoring and pinpoint senescence related cell type (15, 21–23), visualized by the “irGSEA” R package (https://chuiqin.github.io/irGSEA/index.html). Functional enrichment analysis via GO and KEGG database in scRNA data was conducted by “ClusterGVis” R package (https://github.com/junjunlab/ClusterGVis). To explore the specific subgroup preferences of CAF subpopulations, we calculated the odds ratios (OR) via the computational method of Zhang et al. (24). A numeric vector, predicting cellular status from least (1.0) to most (0.0) differentiated was generated from the RNA matrix by “CytoTRACE” R package (25). The “slingshot” and “Monocle3” R package was utilized to infer cell lineages and pseudotime states (26, 27).

### Spatial transcriptomics analysis

To explore biological landscapes of CAFs in pan-cancer spatial transcriptomics (ST) resolution, we sourced ST slide data of various cancer types form 10x database (https://www.10xgenomics.com/cn/datasets), as well as GSE176078, GSE179572, GSE203612 and GSE181300 from GEO database. To accurately acquire the cell composition at each spot on the 10x ST slide, deconvolution analysis was applied (28), which is based on ST and scRNA-seq data, with particular consideration given to the corresponding tumor type. We first obtained scRNA-seq data of various samples with the same tumor type in Tumor Immune Single-cell Hub 2 (TISCH2) (29), and then constructed a comprehensive scRNA-seq reference library. Ensuring the reliability of analysis, we applied strict quality control measures to the scRNA-seq data according to numbers of expressed genes, counts of UMI, and percentage of mitochondrial RNA. In terms of screening parameters, we refer to the relevant studies of sRNA-seq data sources to ensure the scientific and accurate screening criteria. We then constructed a signature score matrix via computing the average expressions of the top 25 specifically expressed genes of various cell types in each site’s scRNA reference. Subsequently, by get_enrichment_matrix and enrichment_analysis in the “Cottrazm” R package (30), we successfully generated the enrichment score matrix, which can provide a powerful support for subsequent cell composition analysis. The enrichment score for each cell type is visualized using the SpatialFeaturePlot function via “Seurat” R package. If the score of Malignant cells in the microregion is 1, the Malignant group is defined. If it is 0, the Normal group is defined; otherwise, the Mixed group is defined. Wilcoxon Rank Sum Test was utilized to assess the statistical difference in gene expression between the three predicted areas. Meanwhile, to obtain the spatial coordinates of CAFs, we performed combined analysis of the scRNA-seq data and ST data via CellTrek R package (31) with its default parameters. We used the run_kdist function to calculate the spatial k-distance between different CAF subtypes and cell subpopulations.

### Establishing models via integrative machine learning algorithms

Utilizing marker genes of senes CAF with prognostic value, we established senes CAF related signature (SCRS) for diagnosing INSS stage 4 NB and predicting prognosis in NB. We unified 10 prognostic machine learning (ML) methods, involving random survival forest (RSF), elastic network (Enet), Lasso, Ridge, stepwise Cox, CoxBoost, partial least squares regression for Cox (plsRcox), supervised principal components (SuperPC), generalized boosted regression modeling (GBM), and survival support vector machine (survival-SVM) for prognosis prediction. Subsequently, 12 diagnostic ML procedures were applied, such as random forest (RF), Lasso, Ridge, elastic net (Enet), stepwise Glm, GlmBoost, LDA, partial least squares regression for Glm (plsRglm), GBM, XGB, SVM and Naives Bayes for the diagnosis of stage 4 NB among all NB patients. A total of 101 prognostic ML methods and 113 predictive ML algorithms were trained within the training cohort, via the leave-one-out cross-validation (LOOCV) framework to establish the prognostic and diagnostic models. Models with fewer than five genes were removed. GSE49710 cohort served as the training set, while GSE85047, TARGET-NB, E-MTAB 8248 and E-MTAB 179 cohorts served as testing sets. The concordance index (C-index) and the average area under the curve (AUC) was calculated in each ML combination across the five sets (13). AUC, Area under precision-recall curve (PRAUC), accuracy, sensitivity, specificity, precision, cross-entropy, Brier scores, balanced accuracy and F1 Score, calculated by “mlr3” R package, were used to select the best diagnostic model (32). We employed precision-recall curve (PRC) to evaluate the performance of classification models in handling imbalanced datasets. Logarithmic loss, recall and decision calibration were utilized to select the best prognostic model, among the top five prognostic models with the highest C-index, via “mlr3proba” R package (33). Risk scores were retrieved with a linear combination method for each prognostic ML combination by including gene expression data from multiple feature selection patterns. Likewise, the most powerful diagnostic model was utilized to calculate the possibility of stage 4 NB.

### Model verification in precision, stability and reliability

Comprehensive validation methods were carried out to validate the superior precision, stability, and replicability of SCRS. With the prognostic SCRS, patients were divided into high or low-risk groups by the median risk score from the training set. Validations was performed based on Kaplan-Meier (KM) survival methods and a log-rank test, with the “survival” and “survminer” R packages. For the diagnostic model, a confusion matrix was utilized to test accuracy of SCRS by “cvms” R package. Receiver Operating Characteristic (ROC) curves, calibration curves and decision curve analysis (DCA) were applied to assess the precision, differentiation, and clinic utility of both diagnostic and prognostic SCRS. Subsequently, we compared the predictive power of the prognostic signature with conventional clinic features in time-dependent ROC curves. Moreover, univariate and multivariate Cox analysis were used to confirm the independent predictive advantage of SCRS.

### Consensus clustering analysis of senescence related CAF makers

With altogether 16 model genes in diagnostic and prognostic SCRS, we performed unsupervised clustering analysis in three NB cohorts (GSE49710, E-MTAB 8248, and TARGET-NB). We used the “ConsensusClusterPlus” R package and the k-means algorithm, which involved 1,000 repetitions and sampled 80% of the total data in each instance, to explore the molecular landscapes of senescent myofibroblast makers (34). Principal components analysis (PCA) was performed to depict the heterogeneity between clusters. The effectiveness of clustering analysis was appraised by comparing differences in clinicopathological characteristics and gene expression levels between clusters, utilizing the “ComplexHeatmap” R package. Moreover, survival analysis was employed to illustrate survival outcome disparities between clusters.

### Function enrichment analysis

Differentially expressed genes (DEGs) were identified from two clusters divided by consensus molecular clustering and from two risk groups categorized by prognostic SCRS. DEGs were discovered with the “limma” R package, by a False-discovery rate (FDR) threshold of <0.05 and an absolute log2fold change (FC) of >1. The function enrichment of DEGs was performed in Gene Ontology (GO) and Kyoto Encyclopedia of Genes and Genomes (KEGG) terms with the “clusterprofiler” R package (35). Gene set variation analysis (GSVA) was conducted by KEGG terms with “GSVA” R package (23), based on the “h.all.v7.4.symbols.gmt” gene set from MSigDB. Gene set enrichment analysis (GSEA) was applied to explore the molecular pathways associated with different clusters and risk groups (36), with a threshold of p < 0.05 and Normalized Enrichment Score (NES) > 1.

### Delineating tumor microenvironment and immune subtypes

We utilized several immune calculation algorithms by the “IOBR” R package and the single sample gene set enrichment analysis (ssGSEA) to assess the immune infiltration levels between groups and clusters by “wilcox” test (37–45). Immune cell marker genes for ssGSEA were sourced from literature (46). Next, the correlation analysis was applied in the Spearman method to reveal the relation between SCRS risk scores, model gene expressions, and immune cell infiltrations. Further, utilizing immune function marker genes, we performed ssGSEA to compare the immune function level between risk groups with “wilcox” test (47). Subsequently, ssGSEA was employed to assess the seven steps of cancer immunity cycle, with marker genes in Tracking Tumor Immunophenotype (TIP) (http://biocc.hrbmu.edu.cn/TIP/) (48). Lastly, we appraised the gene expression levels of immune checkpoint genes in two risk groups. We conducted immune subtype analysis which could identify immune response subtypes and predict immunotherapy reactions (49). Five immunological patterns were revealed in GSE49710 cohort, covering wound healing (C1), IFN-gamma dominant (C2), inflammatory (C3), lymphocyte depleted (C4) and TGF-β dominant (C6). We then compared the proportions of immunological subtypes between groups and clusters.

### Mutational landscape between clusters and groups

Obtaining the somatic mutational data in cBioPortal database (https://www.cbioportal.org/), we appraised the mutational types and frequencies of SCRS model genes with “maftools” R package (50). Moreover, we analyzed the tumor mutation burden (TMB) via calculating the aggregate count of somatic mutations per megabase (MB) within the exonic region of the human genome. Gene mutations were stratified into two types namely synchronous or nonsynchronous mutation. The latter comprised Frame_Shift_Del, Frame_Shift_Ins, In_Frame_Del, In_Frame_Ins, Missense, Nonsense, Nonstop, Splice_Site, and Translation_Start_Site aberrations. Key mutation regions were identified from the copy number variation (CNV) data in cBioPortal database by GISTIC 2.0 (51). Besides, the gene frequency of somatic CNV was visualized in “bubble plot”, while the chromosomal location of gene mutation was visualized in “circle plot” via “RCircos” R package (52).

### Evaluation of immunotherapy and chemotherapy responsiveness

To assess effects of SCRS to forecast immunotherapy response, we compared immune dysfunction and exclusion (TIDE, http://tide.dfci.harvard.edu/) scores between risk groups. Subsequently, we utilized submap algorithm to evaluate immunotherapy responsiveness of patients based on an immunotherapy cohort (53, 54). Meanwhile, we assessed the power of SCRS to predict responses to immunotherapy in immunotherapy datasets (IMvigor210, GSE78220, GSE135222, and GSE91061). Lastly, we acquired the sensitivity to chemotherapy drugs of human cancer cell lines in Cancer Therapeutics Response Portal (CTRP, https://portals.broadinstitute.org/ctrp) database and Profiling Relative Inhibition Simultaneously in Mixtures (PRISM, https://depmap.org/portal/prism/) database. Cell line which is more responsive to a chemotherapy agent could get a lower AUC, which help explore potential therapeutics for high-risk patients (55).

### Single cell analysis of model genes

Six scRNA scoring algorithms were utilized to assess the specific enriched cells of SCRS model genes. We utilized the ‘Scissor’ R package to pinpoint the particular cell populations responsible for SCRS status variations (56), which capitalizes on both aggregate data and phenotypic data, enabling the autonomous selection of cell subpopulations from single-cell datasets, attributing to distinct phenotypes. Pseudotime trajectory analysis was conducted by “Monocle” and “Monocle3” R package, depicting maps of development trajectories devoid of preexisting knowledge about differentiation commencement (27, 57). We used “InferCNV” R package to determine CNVs of neuroendocrine (NE) cells, Schwann cells, endothelial cells, and fibroblasts, referred to T cells, B cells, monocytes and macrophages (58). “CellChat” and “NicheNet” R package was utilized to explore intercellular communication networks in high-SCRS and low-SCRS cells, respectively (59, 60). Subsequently, we utilized “pySCENIC” (version 0.11.2) with Python (version 3.7) to explore enriched transcription factor (TF) and regulon activities of each cell type, which build TF regulatory network and identify steady cell state (61).

### Pan-cancer analysis of hub gene

We performed pan-cancer analysis by “TCGAplot” R package to reveal the commonalities and disparities in genomic and cellular modifications of SCRS hub gene across various cancer types, focusing on gene expression, tumor mutation burden (TMB), microsatellite instability (MSI), immunological microenvironment, and prognosis value (62). We compared gene expression in tumor and normal samples by “wilcox” test and paired samples t-test. Spearman correlation analysis was performed to reveal associations between expression of hub gene and immune infiltrations. Immune cell ratio data was obtained in The Immune Landscape of Cancer (https://api.gdc.cancer.gov/data/b3df502e-3594-46ef-9f94-d041a20a0b9a), and immune score was calculated by ESTIMATE method.

### Immunohistochemistry staining

To verify the different expressions of JAK1 between stage 4 and other NB samples, as well as its prognostic value, we performed immunohistochemistry (IHC) staining in tissue samples from 24 tissue specimens of stage 4 NB and 16 NB tissue specimens of other stages. The study was approved by the ethics committee of Children’s Hospital of Chongqing Medical University. NB tissues were paraffin-embedded and sectioned into 4 mm slices. After dewaxing, hydration, and antigen retrieval, the samples were incubated overnight at 4°C with primary antibody: Anti-JAK1 (Proteintech, Cat No: 66466-1-Ig). Subsequent steps included incubation with a Goat anti-Rabbit IgG secondary antibody (ZENBIO, China), DAB staining (ZENBIO, China), and blocking. Staining was observed under a microscope. Each sample was evaluated for staining intensity (0: none, 1: mild, 2: moderate, 3: strong) and the percentage of positive cells (0: 0%, 1: 1–25%, 2: 26–50%, 3: 51–75%, 4: 76–100%). The final IHC score was the sum of intensity and percentage scores.

## Results

### Establishment of pan−cancer single cell transcriptome atlas of CAFs

**Figure 1:**
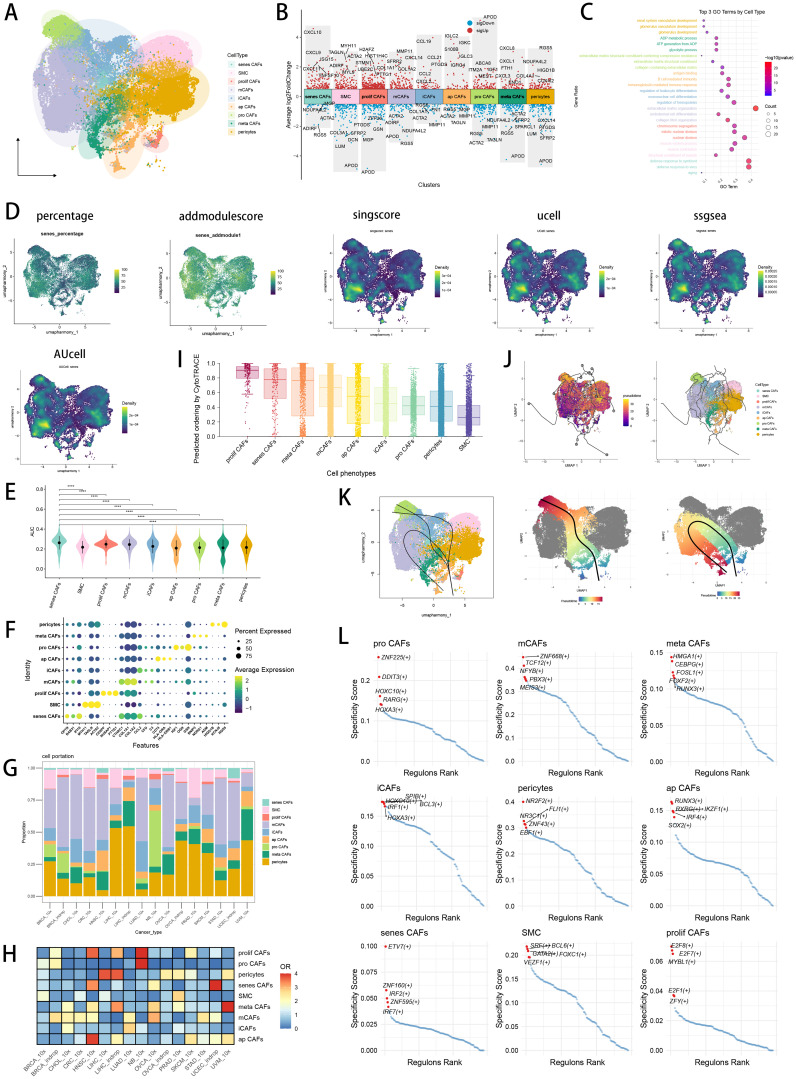
CAF heterogeneity in pan-cancer and identification of senescence related CAFs. **(A)** Visualizing the distribution of CAF subpopulations by UMAP plot in pan-cancer landscape. **(B)** Visualizing each CAF subtype’s marker genes by Manhattan map. **(C)** GO enrichment analysis showed each CAF subtype’s top 3 functional terms. **(D)** Six scRNA scoring algorithms visualized the senescence enriched scores of each CAF subpopulations by UMAP plot. **(E)** AUCell scoring algorithms indicated that senescence related CAFs had the highest senescence score. *p < 0.05; **p < 0.01; ***p < 0.001; ****p < 0.0001. **(F)** Visualizing marker genes of each CAF subtype by dotplot. **(G)** Cell type proportions of each CAF subpopulation across several cancer types. **(H)** Heatmap showing the ORs of CAF subtypes in each cancer type. **(I)** CytoTRACE scores were visualized with boxplot. **(J)** Monocle3 were used to perform pseudotime analyses to infer cellular differentiation states in CAF subpopulations. **(K)** Slingshot were used to perform pseudotime analyses to infer dynamics of lineage specification. **(L)** Scatter plot showing the regulon specificity scores (RSSs) in each CAF subtype via SCENIC analysis. The top 5 regulons are highlighted.

To construct a pan-cancer single-cell and spatial transcriptomics landscape, we downloaded pan-cancer scRNA-seq data from GEO database of 13 prevalent cancer types, along with ST data from 10x website and GEO database. The cancer types of scRNA-seq data that we retrieved from database involved: breast cancer (BRCA), colorectal cancer (CRC), liver hepatocellular carcinoma (LIHC), lung adenocarcinoma (LUAD), cholangiocarcinoma (CHOL), ovarian cancer (OVCA), prostate adenocarcinoma (PRAD), head and neck squamous cell carcinoma (HNSC), neuroblastoma (NB), skin cutaneous melanoma (SKCM), stomach adenocarcinoma (STAD), uveal Melanoma (UVM) and uterine corpus endometrial carcinoma (UCEC) (**Supplementary Figure S1B**). To diminish the batch effects among different scRNA-seq datasets, we independently analyzed each dataset and annotated cells with canonical markers of major cell types. We extracted CAFs from each scRNA-seq dataset and merged them into a Seurat object to establish an integrated scRNA-seq dataset of pan-cancer CAFs. After quality control (**Supplementary Figure S1C**) and batch effect correction by Harmony (**Supplementary Figure S1D**), a total of 35,048 cells in the pan-cancer scRNA-seq data were remained for the following analysis. We next explored the heterogeneity of CAFs in pan-cancer landscape and identify seven CAF subpopulations, as well as pericytes and smooth muscle cells (SMCs) (**Figure 1A**). To evaluate Harmony for batch effect removement, we calculated local inverse Simpson’s Index (LISI) of four batch correction methods, indicating well batch correction after Harmony, as well as the superior ability of batch correction in Harmony algorithm (**Supplementary Figure S1E**).

### CAF heterogeneity and identification of senescence related CAF in pan−cancer

In pan-cancer CAF atlas (**Figure 1A**), we annotated the CAF subpopulation which highly expressed chemokines (CCL19, CCL21 and CXCL2, **Figure 1B**) as inflammatory CAF (iCAF), likely to the previously reported iCAFs (63). A CAF subpopulation which highly expressed extracellular matrix (ECM) remodeling genes (MMP11, CTHRC1 and COL1A2, **Figure 1B**) was annotated as matrix CAF (mCAF), resemble the previously reported mCAFs in cancer data (64). To reveal the molecular landscape of senescence in CAF, we utilized six scRNA scoring algorithms and senescence geneset to conduct enrichment scoring, investigating senescence related CAF subset with the highest enrichment scores of senescence-related genes (**Figures 1D, E**). Finally, we discovered senescence related CAF (senes CAF) subset which ranked first in senescence enrichments scores among all CAF subpopulations (**Figure 1E**; **Supplementary Figure S1F**), as well as highly expressed senescence related genes retrieved from MSigDB database (OPTN, RAB31 and IFI16, **Figure 1F**). Moreover, we found a CAF subset highly expressed MHC-II-associated antigen presentation genes (HLA-DRA, HLA-DRB1 and CD74, **Figure 1F**), which we annotated it as antigen presenting CAF (ap CAF) (65). Then, we observed a CAF subpopulation which was associated with glycolytic process and ATP generation from ADP in GO BP terms (**Figure 1C**), annotating it as metabolic CAF (meta CAF) which was similar to previous research (66). Subsequently, a CAF subpopulation which highly expressed cycle-related genes (CENPF, NUSAP1, and PTTG1, **Figure 1F**) was annotated as proliferative CAF (prolif CAF), consistent with previous pan-cancer research (67). Besides, we annotated progenitor like CAF (pro CAF) subtype with marker genes of IGF1, OGN and GSN (**Figure 1F**) (68). We utilized top marker genes of each CAF subtype to perform GO (**Figure 1C**) and KEGG (**Supplementary Figure S1G**) functional enrichment analysis, which further verified our CAF annotation results. In pan-cancer CAF landscape, we found different CAF subtypes displayed significantly different preferences of cancer types (**Figures 1G, H**). We found that iCAFs and mCAFs were most abundant in CAF subpopulations, while iCAFs were preferred in LUAD, whereas mCAFs were preferred in OVCA (**Figures 1G, H**). And we observed that senes CAFs, were preferred in UCEC and HNSC (**Figure 1H**). The diverse cellular characteristics of CAF subtypes stem from their varied origins, including both tissue-resident fibroblasts and pericytes (69). We utilized CytoTRACE analysis to discover that prolif CAFs owned highest CytoTRACE scores, inferring a possibility to occur in the earlier state of prolif CAFs (**Figure 1I**). With prolif CAFs set as the start point, we used Monocle3 (**Figure 1J**) and slingshot (**Figure 1K**) respectively to conclude differentiation lineages and pseudotime scores among CAF subtypes, revealing the complexity and heterogeneity of CAF differentiation. Meanwhile, we explored the differentially essential motifs within the subtypes of CAFs through SCENIC analysis (**Figure 1L**), with TCF12 motif associated with ECM remodeling ranked highly in mCAFs (70), as well as interferon regulatory factors (IRF) family associated with inflammaging enriched in senes CAFs (71).

### Pan-cancer spatial distribution characteristics of CAF subtypes

**Figure 2:**
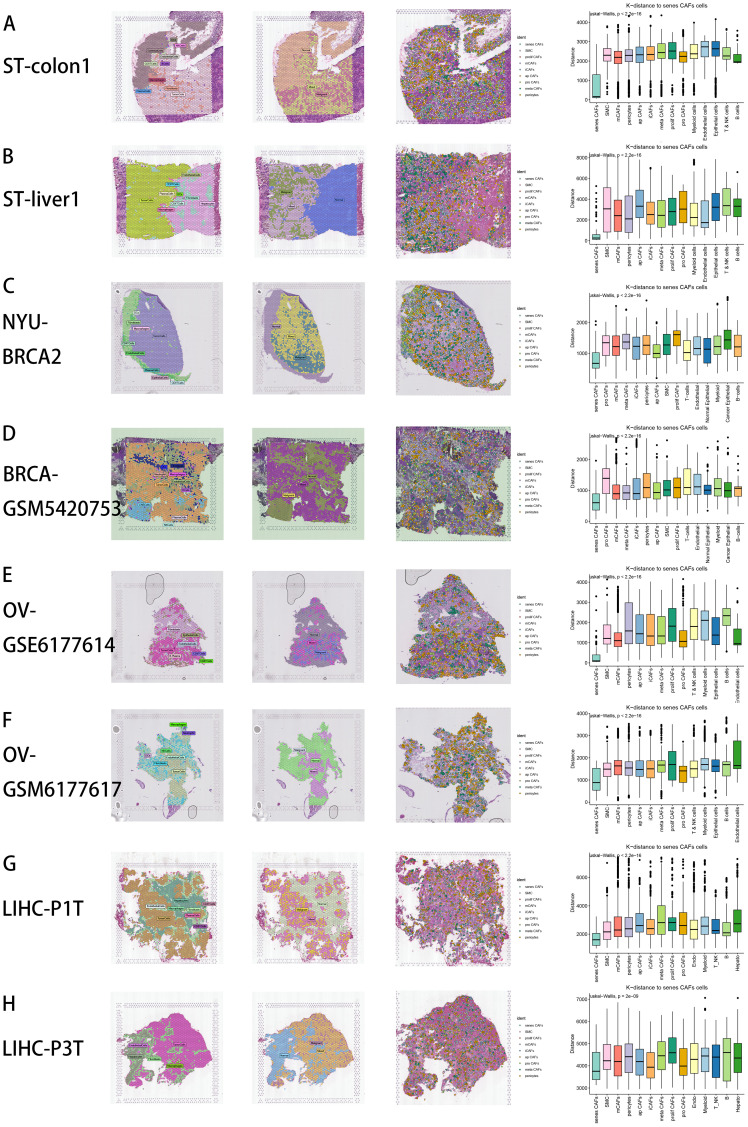
Spatial distribution characteristics of CAFs in CRC **(A)**, CRC liver metastasis **(B)**, BRCA **(C)**, BRCA brain metastasis **(D)**, OV **(E, F)**, LIHC **(G, H)**. From left to right: First: Spatial distribution of various major cell types by RCTD deconvolution analysis in tumor tissue section. Second: Spatial characteristics of malignant areas, mixed areas and normal areas divided by Cottrazm analysis. Third: Spatial cell charting of CAFs in each tissue section using CellTrek. Fourth: CellTrek calculated the average k-distance from different cell types to senes CAFs in each tissue slice.

CellTrek is a computational tool designed to map individual cells directly to the corresponding spatial positions in tissue sections by integrating scRNA-seq and ST data (31). Unlike traditional ST deconvolution techniques, this method transfers ST coordinates to single cells, enabling resolution at the single-cell level. We utilized CellTrek on high-quality scRNA-seq and ST datasets across various cancer types to reconstruct spatial single-cell atlases, involving CRC and CRC liver metastasis (72), as well as BRCA (73), BRCA brain metastasis (74), OVCA (73) and LIHC (75) (**Figures 2A–H**). Remarkably, even in the absence of corresponding scRNA-seq data from the same patient, the ST datasets were largely represented by scRNA-seq data through co-embedding analysis (**Supplementary Figure S1H**). From left to right, the first figure depicts the spatial distribution of various cell types by RCTD deconvolution analysis in tumor tissue section (**Figures 2A–H**). The second figure displays the spatial characteristics of malignant areas, mixed areas and normal areas divided by Cottrazm analysis, indicating the spatial distribution of tumor border and tumor immune barrier in tumor tissue section (**Figures 2A–H**). The third figure reveals the spatial positions of CAF subtypes in tissue sections by CellTrek mapping analysis, which demonstrates an abundant infiltration of various CAF subpopulations and spatial existence of senes CAFs in tumor microenvironment (**Figures 2A–H**). For the fourth figure, we calculated the spatial k-distance between all major cell types and all CAF subtypes in every tumor tissue section, suggesting that CAF subpopulations exhibit the minimum spatial k-distance among themselves (**Figures 2A–H**). For major cell types, we observed that senes CAF exhibit close spatial k-distance to endothelial cells, especially in CRC liver metastasis (**Figure 2B**) and OVCA (**Figure 2E**), while senes CAF also show close spatial k-distance to B cells in CRC (**Figure 2A**) and LIHC (**Figure 2G**).

### Single-cell analysis of major cell types in NB

**Figure 3:**
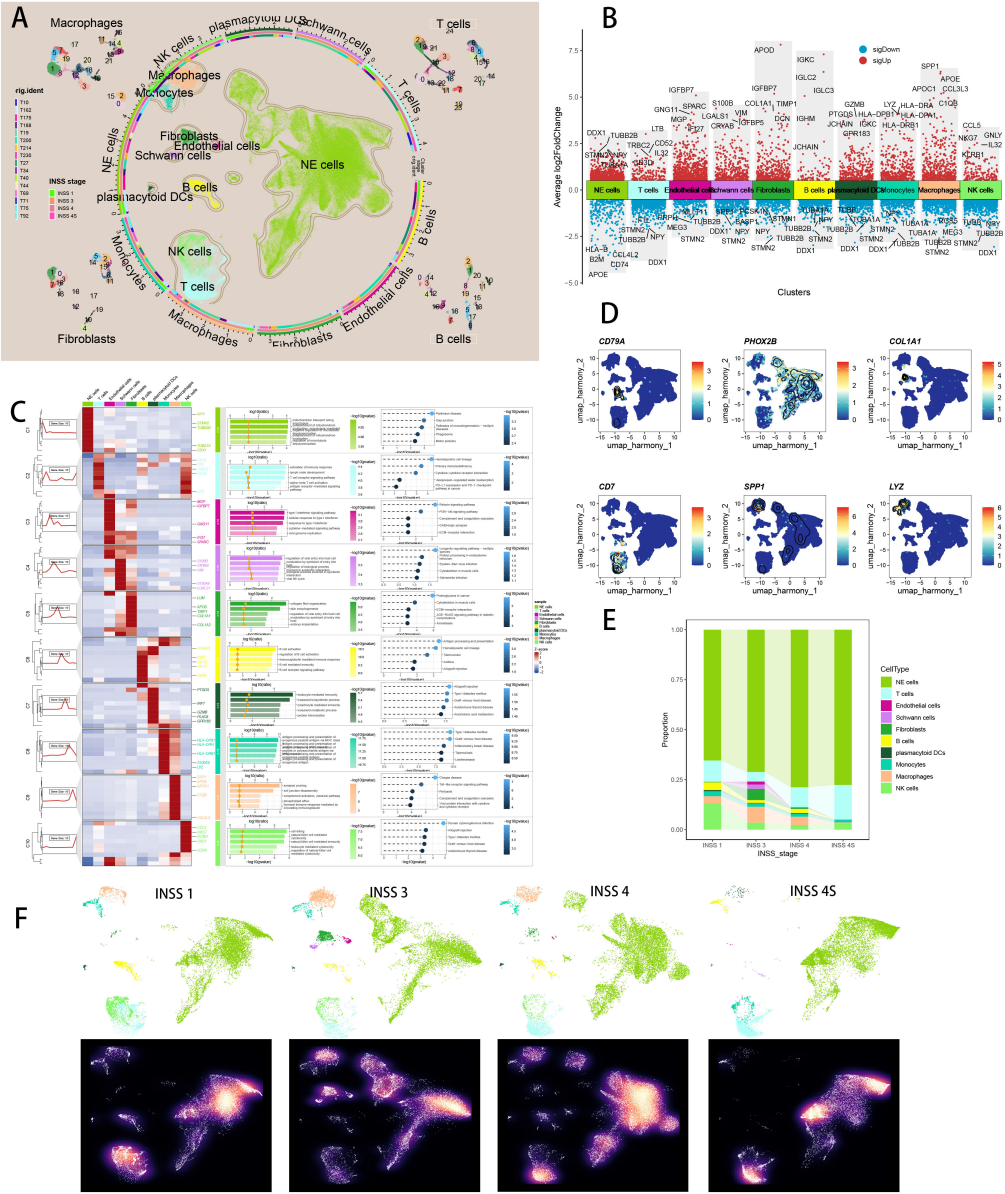
Single cell sequencing analysis of NB tumor samples. **(A)** Visualizing the distribution of 10 major cell populations as well as subpopulations of four major cell types in the TME by UMAP plot. **(B)** Visualizing top 5 marker genes of each major cell type by Manhattan map. **(C)** Visualizing marker genes of each major cell type by heatmap, as well as enrichment analysis results of each major cell type by GO and KEGG. **(D)** Visualizing six representative marker genes of major cell types by UMAP plot. **(E)** The different cell proportions in different INSS stages. **(F)** UMAP view of each cell subsets (top) and cell density (bottom) in different INSS stages.

In the GSE137804 scRNA cohort, we finally retrieved 172,564 cells after proper quality control (**Supplementary Figure S2A**). We performed “harmony” algorithm for batch effect removement, with a fine correction before and after integration (**Supplementary Figure S2B**). Based on cell markers in literature, we manually annotated 10 major cellular subtypes, including NE cells, T cells, B cells, NK cells, endothelial cells, macrophages, monocytes, Schwann cells, fibroblasts, and plasmacytoid DC cells (**Figure 3A**), with subpopulation of macrophages, fibroblasts, B cells and T cells illustrated respectively (**Figure 3A**). **Figure 3B** depicted top 5 marker genes of every major cell type. We visualized top marker genes of each major cell type in heatmap (**Figure 3C**), and utilized GO and KEGG terms to functionally annotated each major cell type, which further verified our single cell annotation (**Figure 3C**). We utilized “Dimplot” to visualize expression levels of several marker genes (CD79A for B cells, PHOX2B for NE cells, COL1A1 for fibroblasts, CD7 for T cells, SPP1 for macrophages and LYZ for monocytes, **Figure 3D**). **Figure 3E** demonstrated the different cell proportions in NB patients of INSS stage 1, 3, 4 and 4S, respectively, with NE cells predominating. **Figure 3F** depicted UMAP view of each cell subsets (top) and cell density (bottom) showing cell distribution across four stages. Downsampling was applied for four tissue groups. High relative cell density is shown as bright magma.

### Single-cell analysis of senescence related CAF in NB

**Figure 4:**
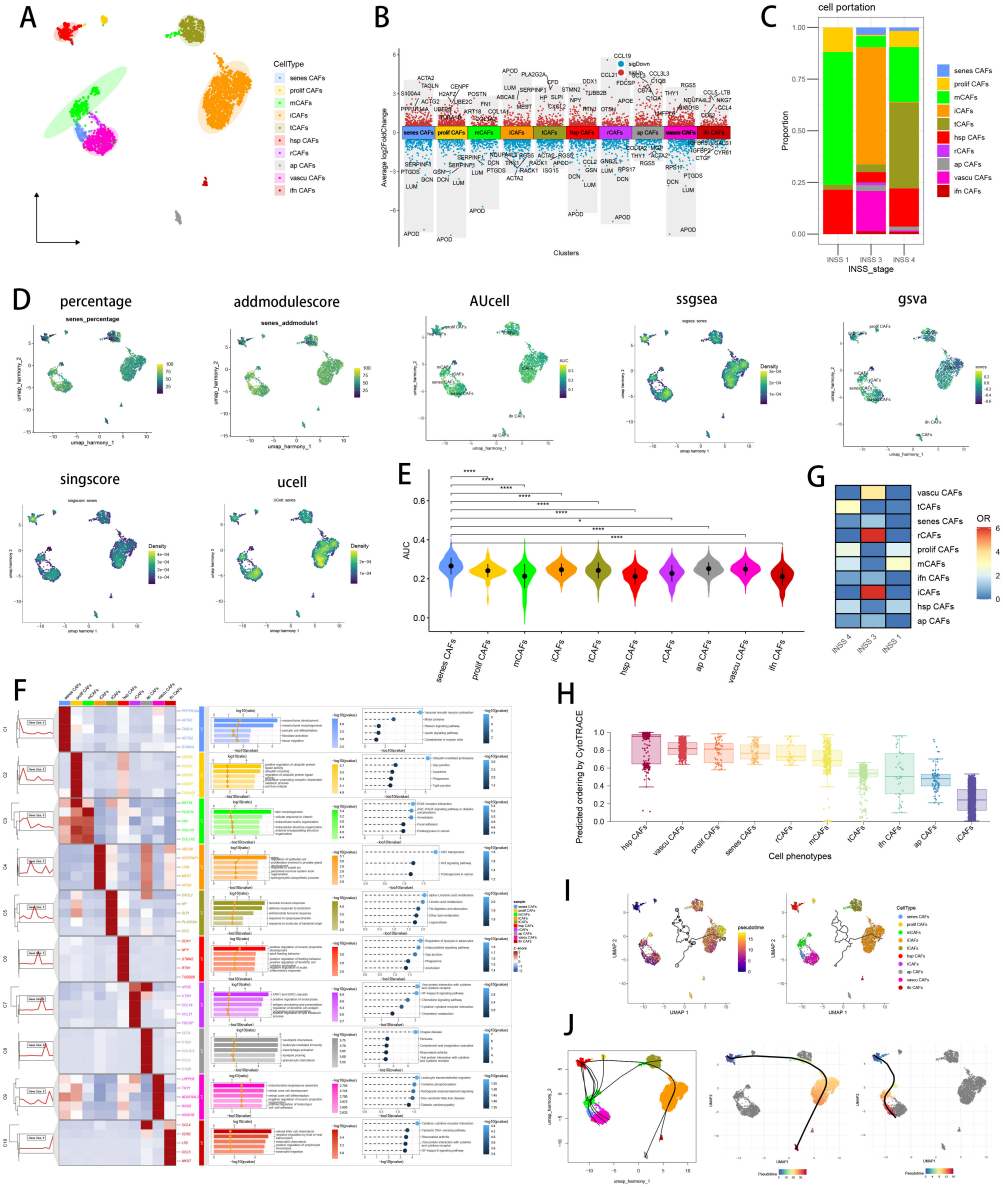
Identification of a distinct CAF subtype namely myofibroblasts which were most associated with senescence. **(A)** Visualizing the distribution of 10 manually annotated CAF subpopulations by UMAP plot. **(B)** Visualizing each CAF subtype’s marker genes by Manhattan map. **(C)** Cell proportion of each CAF subtype in different INSS stages. **(D)** Seven scRNA scoring algorithms visualized the senescence enriched scores of each CAF subpopulations by UMAP plot. **(E)** AUCell scoring algorithms indicated that myofibroblasts had the highest senescence score. *p < 0.05; **p < 0.01; ***p < 0.001; ****p < 0.0001. **(F)** Visualizing marker genes of each CAF subtype by heatmap, as well as enrichment analysis results of each CAF subtype by GO and KEGG. **(G)** Heatmap showing the ORs of CAF subtypes in each INSS stage. **(H)** CytoTRACE scores were visualized with boxplot. **(I)** Monocle3 were used to perform pseudotime analyses to infer cellular differentiation states in CAF subpopulations. **(J)** Slingshot were used to perform pseudotime analyses to infer dynamics of lineage specification.

We subset 3105 fibroblasts and created a new Seurat object for the following scRNA analysis. **Supplementary Figure S2C** showed a well quality control of fibroblast subtypes and **Supplementary Figure S2D** displayed a fine correction of batch effects with Harmony. Based on CAF markers retrieved from literature review (20, 68), we manually annotated 10 CAF subtypes in NB single cell data, namely myofibroblasts (ACTA2, TAGLN, MYH11), proliferative CAF (MKI67), matrix CAF (POSTN, COL3A1), inflammatory CAF (CFD, C3), tumoral CAF (TMEM158), heat-shock protein CAF (HSP90AA1), reticular CAF (CCL19, CCL21), antigen-presenting CAF (CD74, HLA-DRA, HLA-DRB1), vascular CAF (NOTCH3), and interferon CAF (IL32) (**Figure 4A**; **Supplementary Figure S2E**). **Figure 4B** depicted top 5 marker genes of every major cell type. **Figure 4C** illustrated different compositions of CAF subtypes in different INSS stages. Previous literature has reported about senescent myofibroblasts that localize near tumor ducts and accumulate with tumor progression (76), so we utilized seven scRNA scoring algorithms to conduct enrichment scoring and discover senescence related CAF subset, which highlighted myofibroblasts closely associated with senescence (**Figure 4D**). Ranking results of single cell scoring confirmed that myofibroblasts were enriched in senescence and could possibly be identified as the senes CAFs (**Figure 4E**; **Supplementary Figure S2F**). We have included additional three independent scRNA-seq datasets of NB in our analysis to discover the consistent findings (**Supplementary Figures S2G, H**). We visualized top marker genes of each CAF subtype in heatmap (**Figure 4F**), and utilized GO and KEGG terms to functionally annotated each cell type, which further verified our annotation (**Figure 4F**). Interestingly, marker genes of iCAFs were enriched in aging, shedding light on its potential associations with senescence. With the FindAllMarkers function, we identified 1088 significant senescence related CAF markers with a criterion of absolute log2 (fold change) > 0.25 and p-value (Padj) < 0.05 (**Supplementary Table S4**). To sum up, we succeeded in identifying senes CAF subtype, namely myofibroblasts, which is significantly related to senescence. In CAF landscape of NB, different CAF subtypes exhibited different preferences of INSS stages (**Figure 4G**). Subsequently, we utilized CytoTRACE and pseudotime analyses to infer cellular differentiation states and dynamics of lineage specification. Heat-shock protein CAF owned highest CytoTRACE scores, with a likelihood to occur in the earlier state (**Figure 4H**). Setting heat-shock protein CAF as the beginning, we then utilized Monocle3 (**Figure 4I**) and slingshot (**Figure 4J**) respectively to infer several differentiation lineages among CAF subtypes, which showed a relatively senescent state of senes CAF. Moreover, we analyzed the differentially essential motifs within the subpopulations of CAFs with SCENIC analysis (**Supplementary Figure S2I**), with motif SRF, highly associated with cellular senescence, expressed in senes CAFs (77).

### Development and validation of diagnostic SCRS

**Figure 5:**
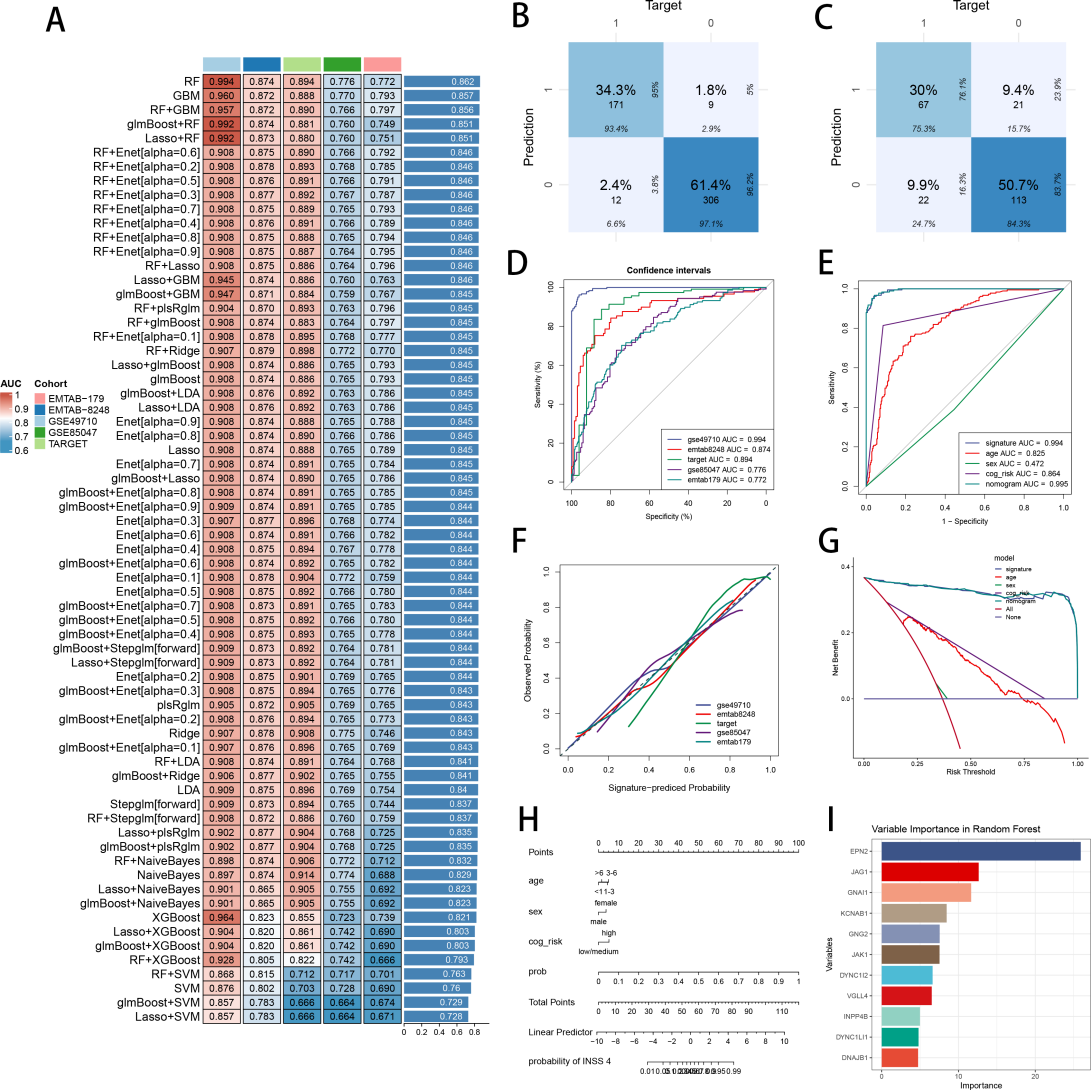
Construction and validation of the diagnostic SCRS to diagnose INSS 4 NB. **(A)** A total of 113 kinds of diagnostic models via a leave-one-out cross-validation framework and further calculated the AUC values of each model. **(B)** Confusion matrix of the diagnostic SCRS in the training cohort GSE49710. **(C)** Confusion matrix of the diagnostic SCRS in the validation cohort E-MTAB 8248. **(D)** ROC curves of the diagnostic SCRS in five cohorts (GSE49710, E-MTAB 8248, TARGET, GSE85047 and E-MTAB 179). **(E)** ROC curves of the diagnostic SCRS, the logistic regression model and clinical variables in GSE49710 cohort. **(F)** Calibration curves of the diagnostic SCRS in five cohorts (GSE49710, E-MTAB 8248, TARGET, GSE85047 and E-MTAB 179). **(G)** DCA curves of the diagnostic SCRS, the logistic regression model and clinical variables in GSE49710 cohort. **(H)** Visualizing the logistic regression model via nomogram. **(I)** The feature importance visualization of 11 variables selected by RF, which formed the final diagnostic model.

We performed batch removements across five NB cohorts, with PCA plots visualizing the well behaviors of batch correction (**Supplementary Figure S3A**). Marker genes of senes CAF were used for feature selection and model development. Ultimately, 13 marker genes with diagnosis significance were involved in the development of diagnostic ML model, while 8 marker genes with prognosis significance were used for establishment of prognostic ML model (**Supplementary Figure S3B**). We performed 101 prognosis ML combinations and 113 predictive ML combinations based on LOOCV framework, to select the best ML combination (**Supplementary Figure S3C**). The model with the highest AUC was established with RF in feature selection and model development, scoring the top average AUC (0.862) in five cohorts (**Figure 5A**). AUC of every ML combination was computed in every test cohort (**Supplementary Table S5**). We utilized Area under precision-recall curve (PRAUC), accuracy, sensitivity, specificity, precision, cross-entropy, Brier scores, balanced accuracy, F1 Score and precision-recall curve (PRC) to reveal that RF model is the best diagnostic model with superior performance (**Supplementary Figures S3D, E**). In GSE49710 cohort (**Figure 5B**) and E-MTAB 8248 cohort (**Figure 5C**), confusion matrix exhibited a fine precision of SCRS with RF model. ROC curves in five datasets displayed a fine discrimination of SCRS (**Figure 5D**). We calculated AUC of SCRS, clinic variable and logistic regression (LR) model including clinic variables and SCRS, showing that SCRS and LR model outperformed (**Figure 5E**). Calibration curves in five datasets displayed well alignments between SCRS predicted possibility and observed possibility (**Figure 5F**), indicates that the model’s probability estimates are reliable and well-calibrated, as it ensures that the risk estimates provided by the model can be trusted to reflect the true likelihood of patient outcomes. DCA curves revealed the great clinical benefit of SCRS and LR model, outperforming other clinic factors (**Figure 5G**). We visualized the diagnostic LR model with nomogram to help clinical decision-making (**Figure 5H**). We obtained feature importance of 11 model genes finally selected by RF, which revealed that hub gene EPN2 was the most powerful in RF algorithm (**Figure 5I**).

### Utilizing prognostic SCRS to predict prognosis in NB

**Figure 6:**
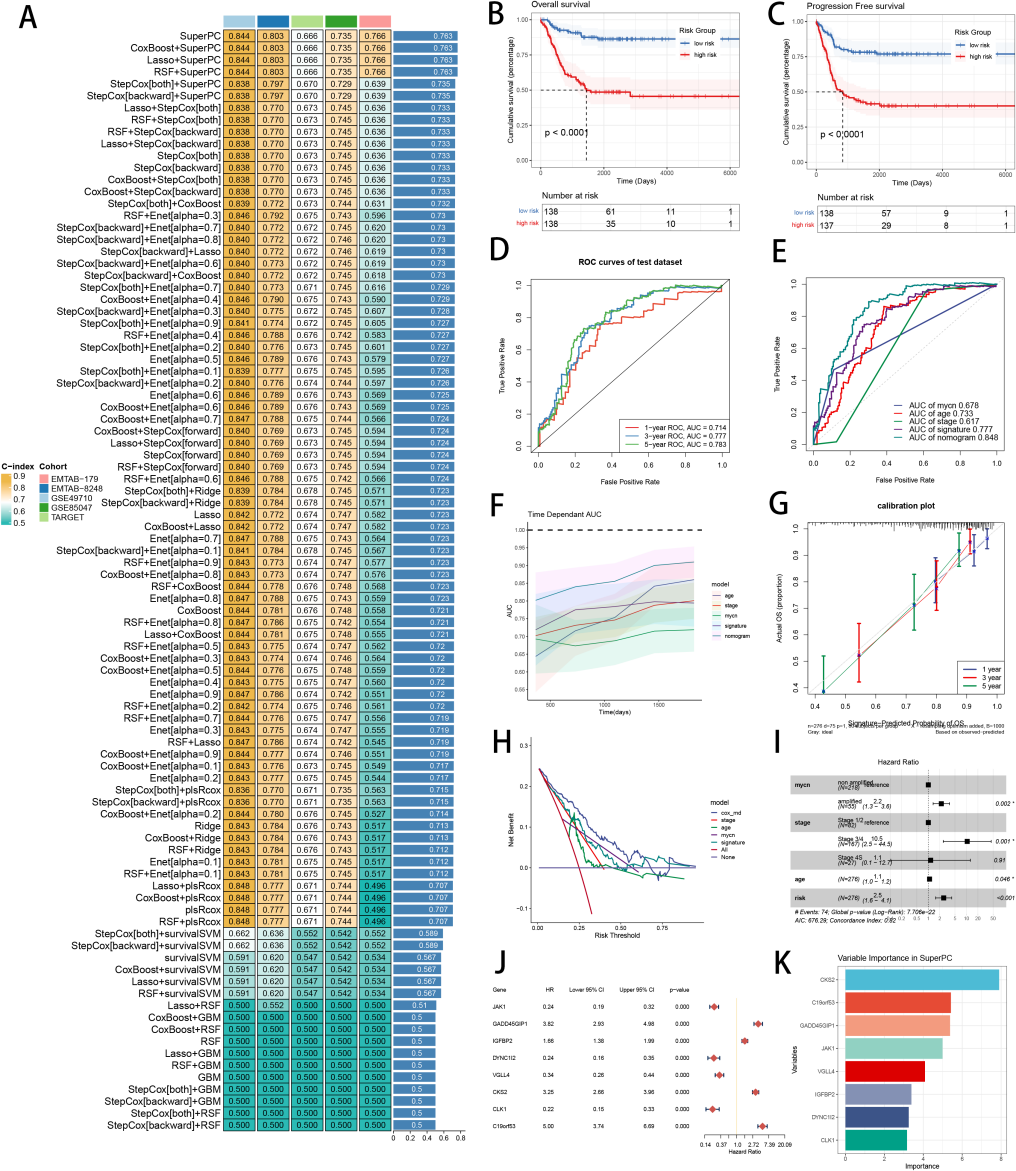
Development and verification of the prognostic SCRS to predict prognosis in NB. **(A)** A total of 101 kinds of prognostic models via a leave-one-out cross-validation framework and further calculated the C-index of each model. **(B)** Kaplan-Meier survival curves of OS for high-risk and low-risk groups of NB patients in GSE85047 cohort. **(C)** Kaplan-Meier survival curves of PFS for high-risk and low-risk groups of NB patients in GSE85047 cohort. **(D)** ROC curves of 1-, 3- and 5-year OS of the prognostic SCRS in GSE85047 cohort. **(E)** AUC values of 3-year OS of the prognostic SCRS, the cox regression model and clinical variables in GSE85047 cohort. **(F)** Time dependent ROC curves of the prognostic SCRS, the cox regression model and clinical variables in GSE85047 cohort. **(G)** 1-, 3- and 5-year calibration curves of the prognostic SCRS in GSE85047 cohort. **(H)** DCA curves of the prognostic SCRS, the cox regression model and clinical variables in GSE85047 cohort. **(I)** Forest plot visualized the outcome of multivariate Cox regression analysis involving the prognostic SCRS and clinical variables. **(J)** Results of univariate cox regression analysis of 8 variables in SCRS. **(K)** The feature importance visualization of 8 variables in SuperPC algorithm.

The ML combination with the highest C-index was constructed by SuperPC in feature selection and model development, obtaining the highest average C-index (0.763) in five cohorts (**Figure 6A**). C-index of every ML combination was computed in every test cohort (**Supplementary Table S6**). Logarithmic loss, recall and decision calibration were computed to reveal that SuperPC model had the best performance in calibration and precision (**Supplementary Figure S3F**). In GSE85047 dataset, the low-risk patients had a longer overall survival (OS) and progression-free survival (PFS) than the high-risk patients (**Figures 6B, C**). ROC curves in 1-, 3- and 5-year OS displayed fine specificity in SCRS (**Figure 6D**). AUC values of 3-year OS indicated that SCRS and cox regression model including SCRS and clinic factors were more discriminative to predict prognosis than other clinic factors (**Figure 6E**). Time dependent ROC curves revealed that SCRS and cox regression model behaved better than common clinic factors in discriminative ability (**Figure 6F**). Calibration curves (**Figure 6G**) and DCA curves (**Figure 6H**) demonstrated that SCRS is powerful in accuracy and clinical benefit, which implies that using the SuperPC model to guide clinical decision-making would result in more effective identification of patients. Multivariate Cox regression analysis indicated that SCRS risk score was an independent prognosis variable in GSE85047 cohort (P < 0.001) (**Figure 6I**). We utilized univariate cox regression to depict the prognosis value of each model gene (**Figure 6J**). We visualized the feature importance of 8 model genes chosen by SuperPC, with CKS2 being the most influential (**Figure 6K**). These metrics of model evaluation consistently proved that SCRS exhibited precision and robustness in model performance.

### Function enrichment and gene expression landscape

**Figure 7:**
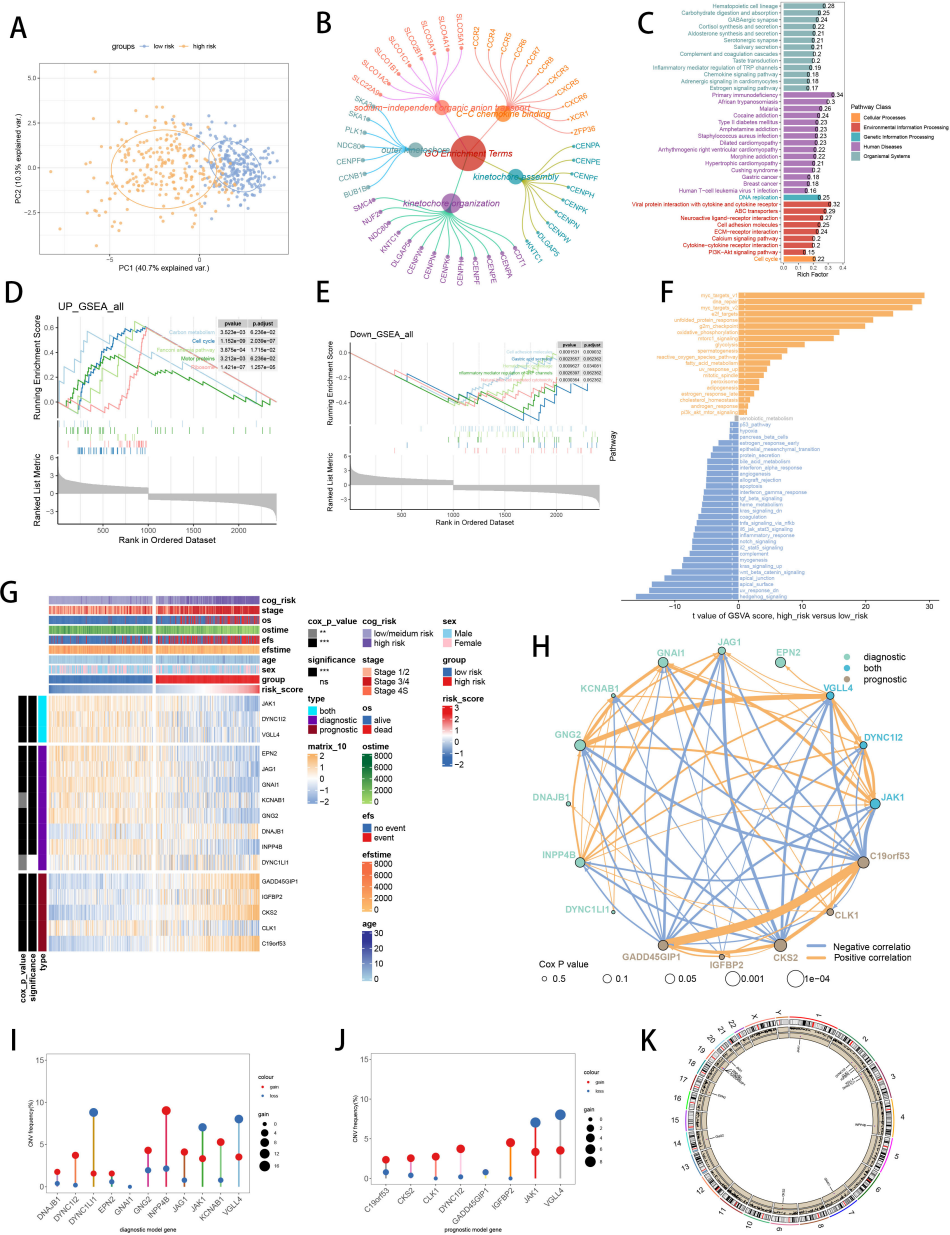
Functional enrichment analysis and landscape of SCRS model genes in GSE49710 cohort. **(A)** PCA analysis plot of high-risk group and low-risk group. **(B, C)** GO and KEGG enrichment analyses of DEGs among two risk groups. **(D-F)** GSEA and GSVA analyses of DEGs among two risk groups. **(G)** Differences in the expression of model genes and differences in the clinical variables of NB patients among the two risk groups. ns: not significant; *p < 0.05; **p < 0.01; ***p < 0.001; ****p < 0.0001. **(H)** Molecular interaction network plot visualized the correlations among expressions of model genes and their prognostic prediction value. Significantly positive and negative correlations are shown as red and blue lines, respectively. The color and size of the nodes indicate the type of model genes and P values from Cox regression. **(I, J)** The CNV mutation frequency of the diagnostic model genes and the prognostic model genes. **(K)** Chromosome position and alteration of all model genes.

Further explorations were undertaken to elucidate the underlying mechanism of SCRS model genes. PCA showed significant division between two risk groups, split by prognostic SCRS (**Figure 7A**). Utilizing DEGs sourced from differential analysis between two risk groups, we employed function enrichment analysis through GO and KEGG databases, which revealed that DEGs were enriched in outer kinetochore in GO terms, and were enriched in DNA replication in KEGG terms (**Figures 7B, C**). GSEA discovered that Ribosome and Motor proteins were elevated in high-risk patients, and cell adhesion molecules were decreased in high-risk patients (**Figures 7D, E**). Then, GSVA with”h.all.v7.4.symbols.gmt” gene set in MSigDB website demonstrated that high-risk patients were elevated in myc_targets_v1, and decreased in hedgehog_signaling (**Figure 7F**). The diverse expressions of SCRS model genes and the variation of clinic factors between two risk groups were obvious (**Figure 7G**). The hub gene JAK1 expressed significantly lower in high-risk patients, exhibiting significant protective prognosis value. Spearman correlation analysis indicated tight relations (correlation p value < 0.001) across SCRS model genes (**Figure 7H**). Utilizing CNV data to plot, we displayed that INPP4B had the highest somatic CNV frequency in diagnosis model genes, and VGLL4 had the highest somatic CNV frequency in prognosis model genes (**Figures 7I, J**). Moreover, we portrayed the loci of mutations within SCRS model genes on the chromosome (**Figure 7K**).

### Implications of SCRS with tumor immune microenvironment

**Figure 8:**
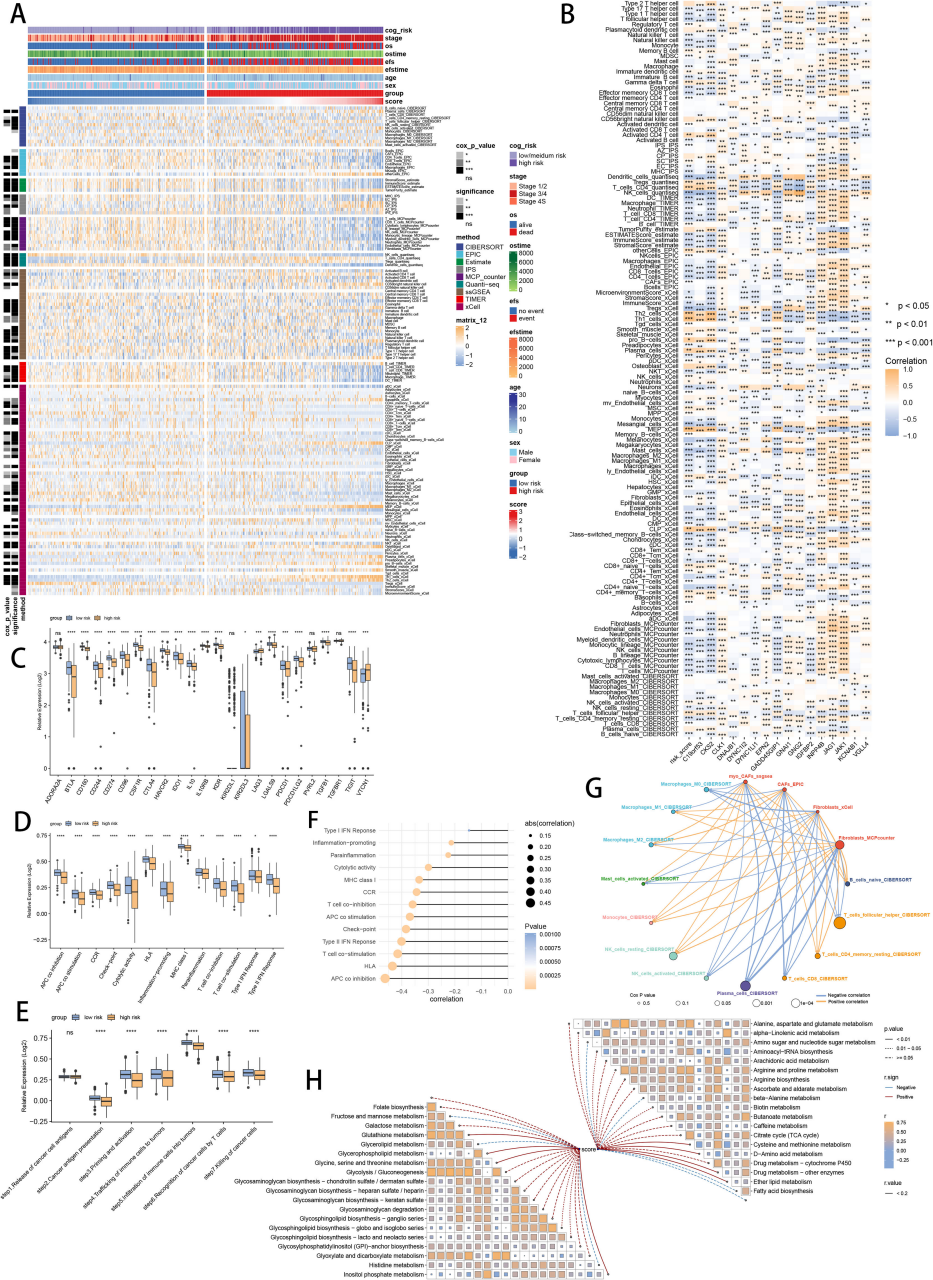
Analysis of the TME in different risk groups in GSE49710 cohort. **(A)** Differences in immune infiltration status between two risk groups were evaluated by eight immune algorithms. ns: not significant; *p < 0.05; **p < 0.01; ***p < 0.001; ****p < 0.0001. **(B)** Heatmap visualized the correlation between different immune cells and risk scores and the relationship between different immune cells and expressions of model genes. **(C)** The differences of expressions of immune checkpoint related genes between two risk groups. **(D)** The differences of immune function scores calculated by ssGSEA analysis between two risk groups. **(E)** The differences of cancer immunity cycle scores based on ssGSEA analysis between two risk groups. **(F)** The correlations between risk scores and immune function scores in bubble plot. **(G)** Molecular interaction network plot visualized the correlations among immune cell and CAFs in TME and their prognostic prediction value. **(H)** The correlations between risk scores and metabolic related pathways based on GSVA analysis of KEGG terms were displayed in butterfly plot.

To assess the discrimination of prognostic SCRS in immune infiltration, we analyzed the immune cell abundance in two groups using eight immune algorithms. We used “ComplexHeatmap” R package to revealed the significantly lower immune cell infiltrations in high-risk patients (**Figure 8A**). Meanwhile, the spearman correlation method showed associations between immune cell abundances and SCRS risk scores, as well as relationships between immune cell abundances and model gene expressions (**Figure 8B**). Additionally, low-risk patients had higher expression profiles of immune checkpoint genes, prompting a sensitivity to immunotherapy (**Figure 8C**). Moreover, ssGSEA based on immunological function signatures revealed that the low-risk group was significantly more infiltrated in immune cells (**Figure 8D**). Besides, ssGSEA results of six key steps in cancer immunity cycle was significantly higher in low-risk patients (**Figure 8E**). The spearman correlation method showed inverse associations between immune function levels and SCRS risk scores (**Figure 8F**). We utilized top 10 maker genes of myofibroblasts and conducted ssGSEA to obtain immune abundance of senescence related myofibroblasts. Then we conducted spearman correlation analysis to reveal tight relations (correlation p value < 0.001) across immune cells and CAFs in TME, as well as their cox p value (**Figure 8G**). Given the well-established diverse metabolic inclinations and dependencies (78), we acquired various metabolic pathways in KEGG terms to explore associations between risk scores and cancer metabolic pathways, which revealed underlying metabolic functions of SCRS (**Figure 8H**).

### Assessment of the gene mutational profiles

**Figure 9:**
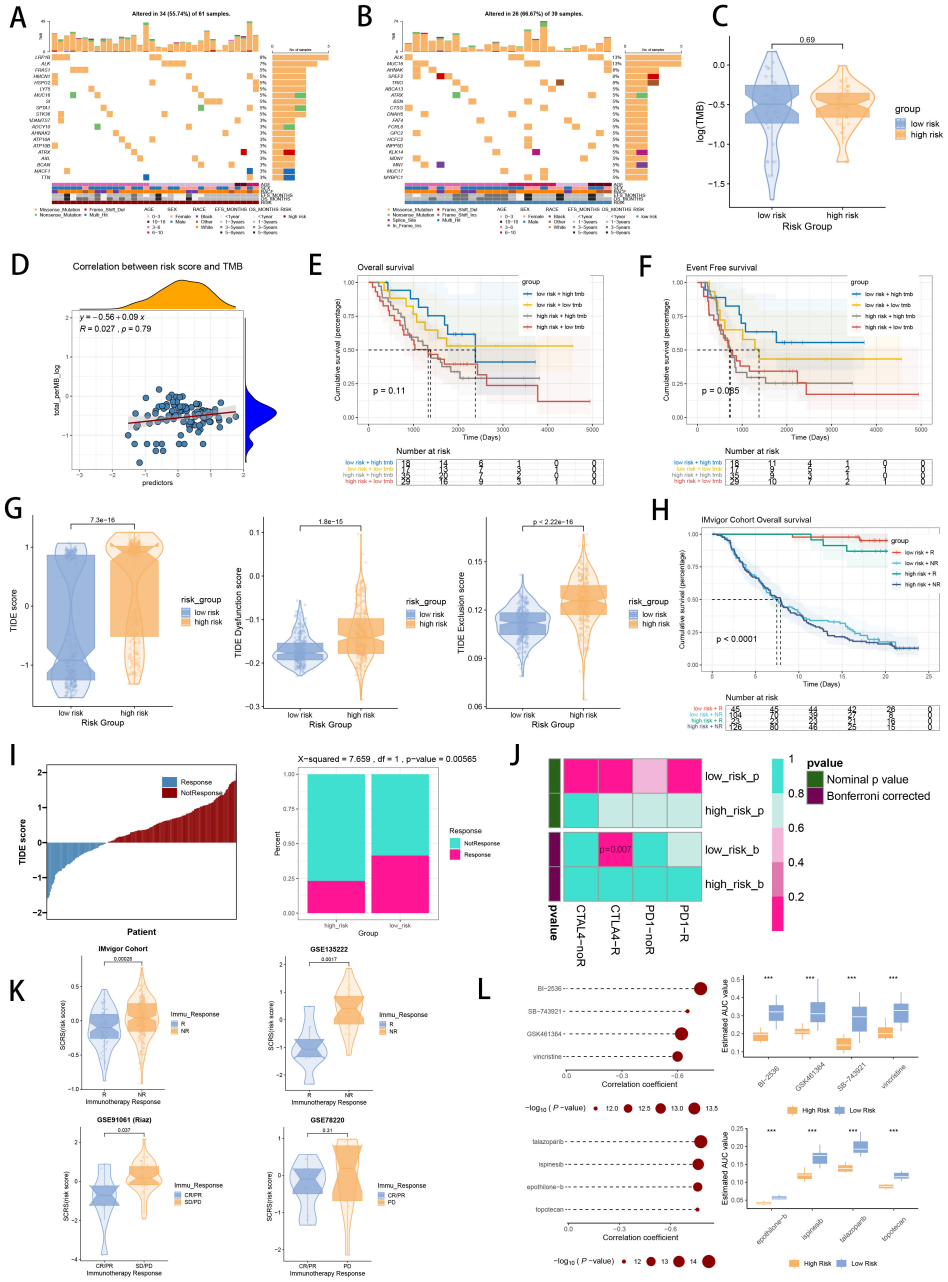
Landscape of somatic mutation, CNVs, immunotherapy and chemotherapy between high-risk and low-risk groups. **(A, B)** Visual summary displayed common genetic alterations in the high-risk and low-risk groups in TARGET cohort. **(C)** Tumor mutation burdens between high-risk and low-risk groups in TARGET cohort. **(D)** Spearman correlation between SCRS risk scores and TMB scores in TARGET cohort. **(E, F)** Comprehensive survival analysis on OS and EFS based on two risk groups and two TMB groups in TARGET cohort. **(G)** Violin diagram illustrated the variance in TIDE scores between high-risk and low-risk groups in GSE49710 cohort. **(H)** Kaplan-Meier survival analysis delineated the OS rates for patients categorized into high-risk and low-risk groups in IMvigor cohort. **(I)** The TIDE algorithm predicted response to immunotherapy between high-risk and low-risk groups in E-MTAB 8248 cohort. **(J)** Comprehensive submap analysis predicted response to immunotherapy between high-risk and low-risk groups in E-MTAB 8248 cohort. **(K)** Box diagram depicted the disparity in SCRS risk scores among immunotherapy patients in the IMvigor210, GSE78220, GSE135222, and GSE91061 immunotherapy cohorts. **(L)** Correlation study and differential drug response analysis of CTRP-derived pharmaceuticals and PRISM-derived pharmaceuticals to explore potential drugs for high-risk NB patients. *p < 0.05; **p < 0.01; ***p < 0.001; ****p < 0.0001.

We demonstrated the landscapes of somatic mutation in high-risk patients (**Figure 9A**) and low-risk patients (**Figure 9B**) with mutational information in TARGET cohort. High-risk patients owned more tumor mutation burden (TMB) than low-risk patients with no significance (**Figure 9C**). Meanwhile, TMB was positively related to SCRS risk scores according to spearman correlation analysis with no significance (**Figure 9D**). Then, we classified patients of TARGET cohort into high TMB and low TMB subgroup based on median TMB. Survival analysis revealed that high-risk patients with low TMB showed the shortest OS and EFS, and low-risk patients with high TMB showed the longest EFS, with significance (**Figures 9E, F**).

### Responsiveness of immunotherapy and potential therapeutic targets

Utilizing TIDE and submap method, the responsiveness of immunotherapy was assessed in high-risk and low-risk patients. In GSE49710 dataset, high-risk group showed higher TIDE scores, higher TIDE dysfunction and exclusion scores, which is prone to exhibit immune escape during immunotherapy (**Figure 9G**). In IMvigor210 dataset, low-risk patients responsive to immunotherapy showed the longest OS, while high-risk patients not responsive to immunotherapy owned the shortest OS, with significance (**Figure 9H**). Meanwhile, in E-MTAB 8248 dataset, low-risk patients were more likely to respond to immunotherapy (**Figure 9I**). Subsequently, submap method revealed that low-risk group were more responsive to CTLA4 inhibitors (p = 0.007) and not responsive to PD1 inhibitors (**Figure 9J**). Subsequently, we compared therapy effects in four immunotherapy datasets between risk groups, which revealed that patients responsive to immunotherapy had lower risk scores, with significance (**Figure 9K**). Ultimately, to discover novel chemotherapy agents for high-risk patients identified by SCRS, we forecasted the medicine reaction utilizing drug sensitivity data sourced in CTRP and PRISM. By cross correlating the two pharmacogenomics datasets, we triumphantly obtained five possible medicines or compounds (BI-2536, GSK461364, SB-743921, ispinesib and talazoparib), which exhibited therapeutic effectiveness in high-risk patients (**Figure 9L**).

### Identifying SCRS model genes associated clusters

**Figure 10:**
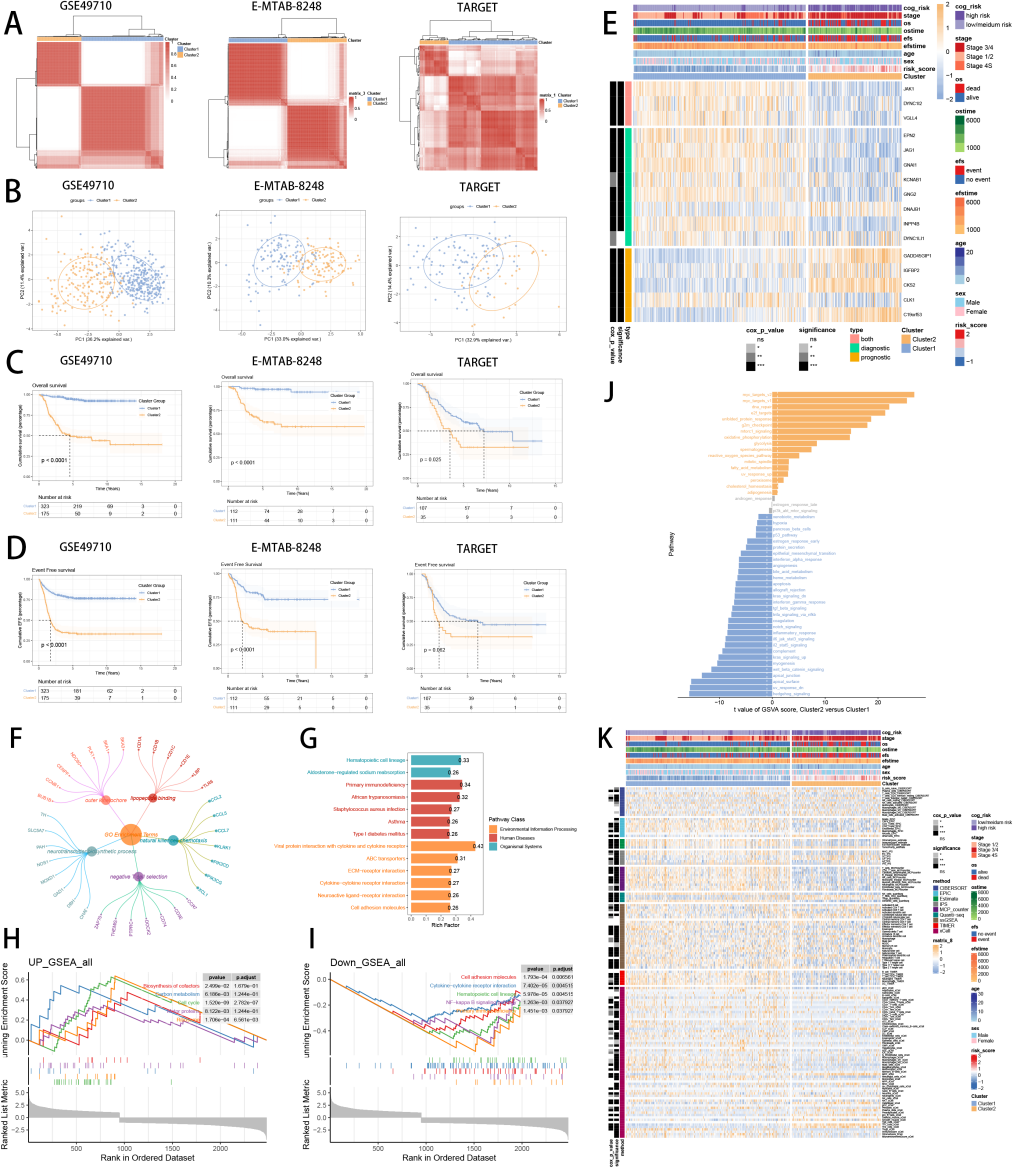
Consensus clustering analysis of SCRS model genes related clusters in three NB cohorts (GSE49710, E-MTAB 8248, TARGET). **(A)** Consensus matrixes of NB patients for k = 2. **(B)** PCA analysis of two clusters. **(C, D)** Kaplan-Meier survival analysis of OS and EFS between two clusters. **(E)** ComplexHeatmap of the distribution of SCRS model genes and clinical variables in the two clusters in GSE49710 cohort. ns: not significant; *p < 0.05; **p < 0.01; ***p < 0.001; ****p < 0.0001. **(F, G)** GO and KEGG enrichment analysis indicated significant enrichment of pathways in cluster 2. **(H-J)** GSEA and GSVA analyses of DEGs among two clusters. **(K)** Differences in the proportion of various kinds of immune cells calculated by eight immune algorithms in two clusters. ns: not significant; *p < 0.05; **p < 0.01; ***p < 0.001; ****p < 0.0001.

To deeply explore the expression profiles of SCRS model genes, GSE49710, E-MTAB 8248 and TARGET-NB were involved to perform consensus molecular clustering. We used SCRS model genes to perform unsupervised clustering analysis in every dataset, which revealed k = 2 with outstanding discrimination (**Figure 10A**). Meanwhile, PCA displayed indispensable disparities between two clusters (**Figure 10B**). Then, survival analysis showed that the cluster 2 had shorter OS (**Figure 10C**) and EFS (**Figure 10D**), with significance. Next, the expression landscapes of SCRS model genes and the clinic variables between two clusters were significantly different (**Figure 10E**). Using DEGs obtained between two clusters, we employed function enrichment analysis to found that DEGs were enriched in negative T cell selection in GO, and were enriched in ABC transporters in KEGG (**Figures 10F, G**). GSEA demonstrated that biosynthesis of cofactors was elevated in cluster 2, and NK-kappa B signaling pathway were decreased in cluster 2 (**Figures 10H, I**). GSVA, with “h.all.v7.4.symbols.gmt” gene set in MSigDB, demonstrated that cluster 2 was elevated in myc_targets_v2, and decreased in hedgehog_signaling (**Figure 10J**). Subsequently, we utilized eight immune infiltration methods to appraise the immunological infiltration variations in two clusters, as well as depicting Cox P value of every cell type (**Figure 10K**).

### Model comparisons and immune subtypes

**Figure 11:**
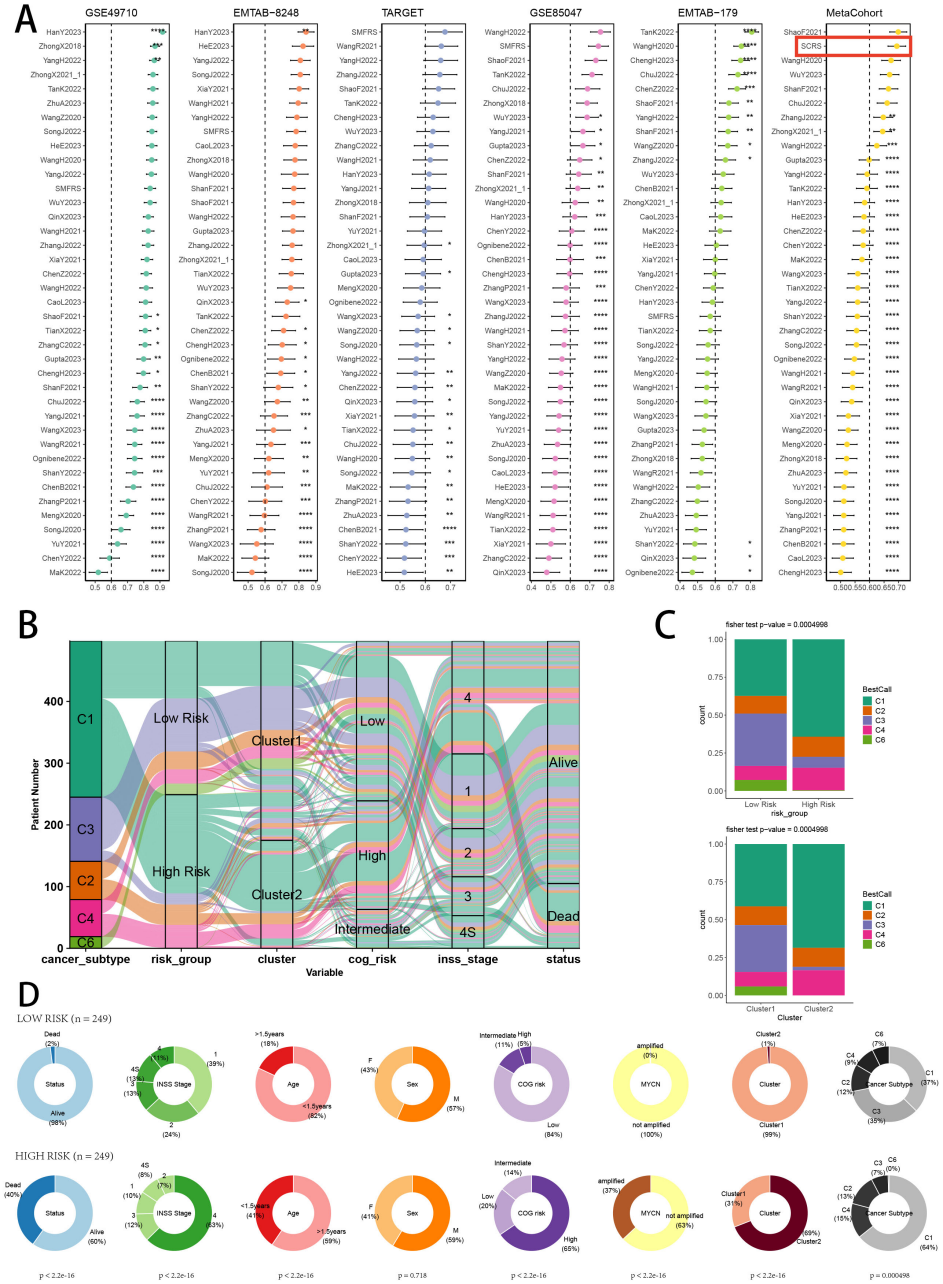
Model comparisons and landscape of two risk groups and two clusters. **(A)** C-index comparison analysis between the prognostic SCRS and 39 published signatures in GSE49710, E-MTAB 8248, TARGET, GSE85047, E-MTAB 179 and meta-cohort. *p < 0.05; **p < 0.01; ***p < 0.001; ****p < 0.0001. **(B)** Sankey diagram of distributions in two clusters and two risk groups with different clinical variables and survival outcomes. **(C)** Differences in the proportion of five immune subtypes between two clusters and two risk groups. **(D)** Circular pie chart visualized the proportion difference of clinical indices and immune subtypes between two risk groups in GSE49710 cohort.

To validate the superior prognostic prediction ability of SCRS, we gathered gene coefficients of 39 released public NB prognosis signatures. Then, we compared C-index of every prognostic signature with SCRS in five NB datasets. Ultimately, we revealed that SCRS outperformed most of previous signatures in five datasets in prediction performances (**Figure 11A**), which qualified SCRS as a meaningful NB prognostic signature. Moreover, we demonstrated the relationships between risk groups, clusters and clinic factors via “sankey plot” (**Figure 11B**). After defining immunological subtypes of patients in GSE49710, we found significant differences of respective proportions of immunological subtypes between risk groups and clusters, indicating more wound healing (C1) subtype in high-risk patients and cluster 2 (**Figure 11C**). Comparisons of clinical factors between two risk groups revealed that low-risk patients showed longer prognoses and better clinic status in GSE49710 cohort (**Figure 11D**).

### Single-cell scoring, scissor algorithm and pseudotime trajectory

**Figure 12:**
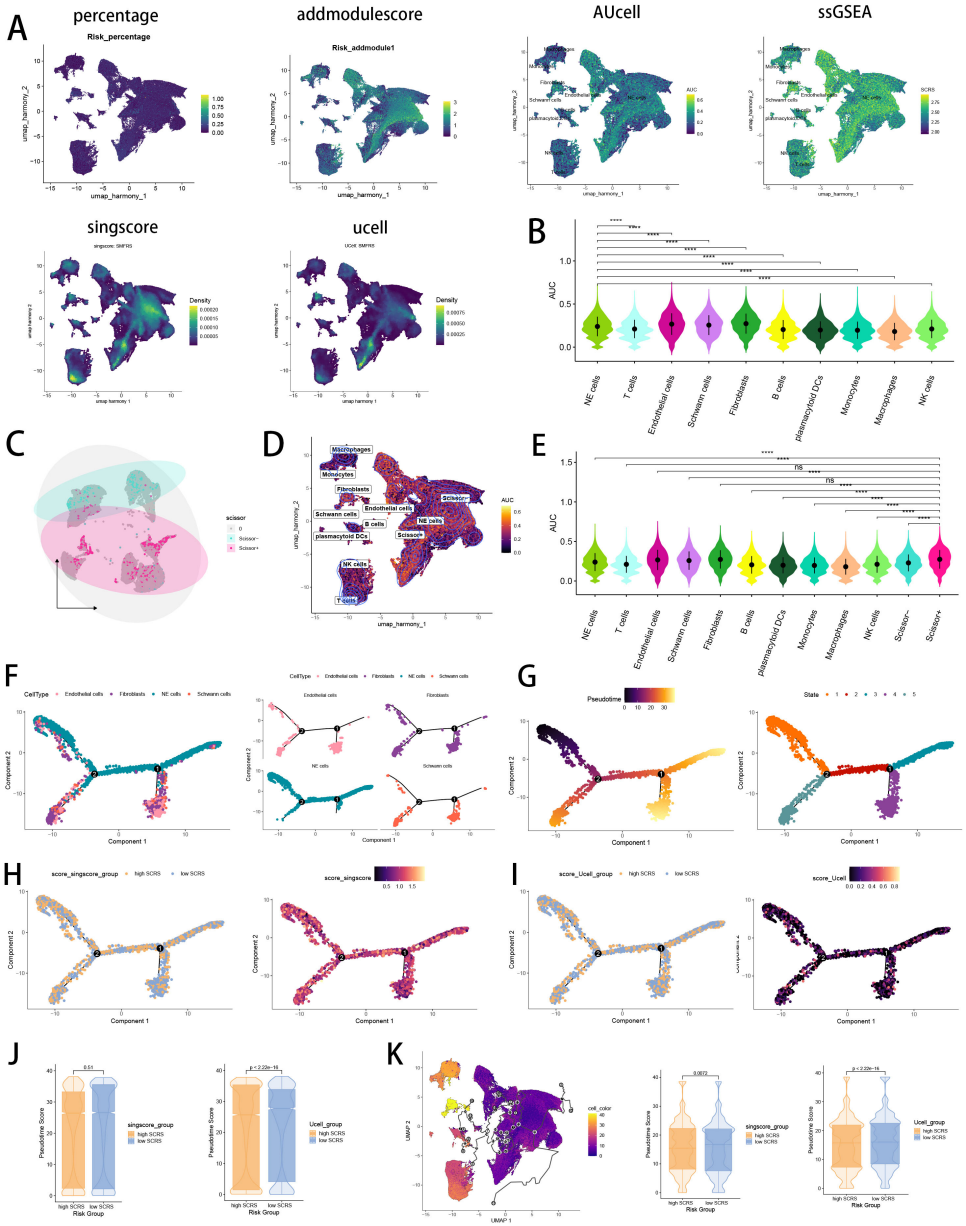
Exploration of SCRS model genes in GSE137804 scRNA-seq cohort. **(A)** Single cell scoring results of SCRS model genes based on six scRNA scoring algorithm in UMAP plot. **(B)** Comparisons of single cell scoring results of SCRS model genes based on AUCell scoring algorithm. ns: not significant; *p < 0.05; **p < 0.01; ***p < 0.001; ****p < 0.0001. **(C)** Visualization of high-SCRS cells (Scissor+ cells) and low-SCRS cells (Scissor- cells) in NB cells. **(D)** Visualization of high-SCRS cells (Scissor+ cells) and low-SCRS cells (Scissor- cells) in UMAP plot with other cell types. **(E)** Comparisons of single cell scoring results of SCRS model genes among Scissor+ cells and Scissor- cells based on AUCell scoring algorithm. ns: not significant; *p < 0.05; **p < 0.01; ***p < 0.001; ****p < 0.0001. **(F, G)** Pseudotime trajectory analysis in NB cells, fibroblasts, endothelial cells and Schwann cells via Monocle 2 algorithm (Cells are colored according to cell types, pseudotime, and states). **(H-J)** Pseudotime trajectory analysis based on Monocle 2 algorithm revealed significant differences of pseudotime scores between high-SCRS cells and low-SCRS cells (Cells are colored in single cell scoring results and high-SCRS or low-SCRS groups). **(K)** Pseudotime trajectory analysis based on Monocle 3 algorithm revealed significant differences of pseudotime scores between high-SCRS cells and low-SCRS cells.

Verifying the risk-stratify ability of SCRS in single cell landscape, we calculated the SCRS enrichment scores via scRNA scoring algorithms, which revealed that higher-SCRS cells were mostly abundant in NE cells (**Figures 12A, B**). To elucidate the cellular origins supporting high-SCRS clinical manifestations, we employed the “scissors” R package to make correlations between bulk-seq data and scRNA data, which independently singles out cells having remarkable alignment with the desired phenotype. We labeled high-SCRS and low-SCRS states of patients as our foremost phenotypes, thus enabling the thorough detection of 2120 high-SCRS cells (scissors+ cells) and 1875 low-SCRS cells (scissors- cells) (**Figures 12C, D**). The SCRS values of scissors+ cells were significantly higher than scissors- cells and other cell types (**Figures 12D, E**), which indicated successful discovery of scissors+ cells representing diverse SCRS status. Subsequently, we performed pseudotime trajectory analysis via Monocle 2 algorithm to explore the temporal sequences of cellular differentiation in NE cells, fibroblasts, Schwann cells and endothelial cells, with part of NE cells being poorly differentiated (**Figures 12F, G**). We utilized SCRS scores calculated by singscore and Ucell algorithm to divided cells into high-SCRS and low-SCRS according to median SCRS score. Comparisons of pseudotime scores revealed that high-SCRS NE cells had an earlier differentiation trajectory than low-SCRS NE cells, which indicated that immature NE cells scored higher SCRS points (**Figures 12H–J**). Additionally, setting NE cells as differentiation starting point in Monocle 3 algorithm, we verified that immature NE cells scored higher SCRS points, which could serve as malignant cells (**Figure 12K**).

### Verification of SCRS via inferCNV, cell communication and SCENIC

**Figure 13:**
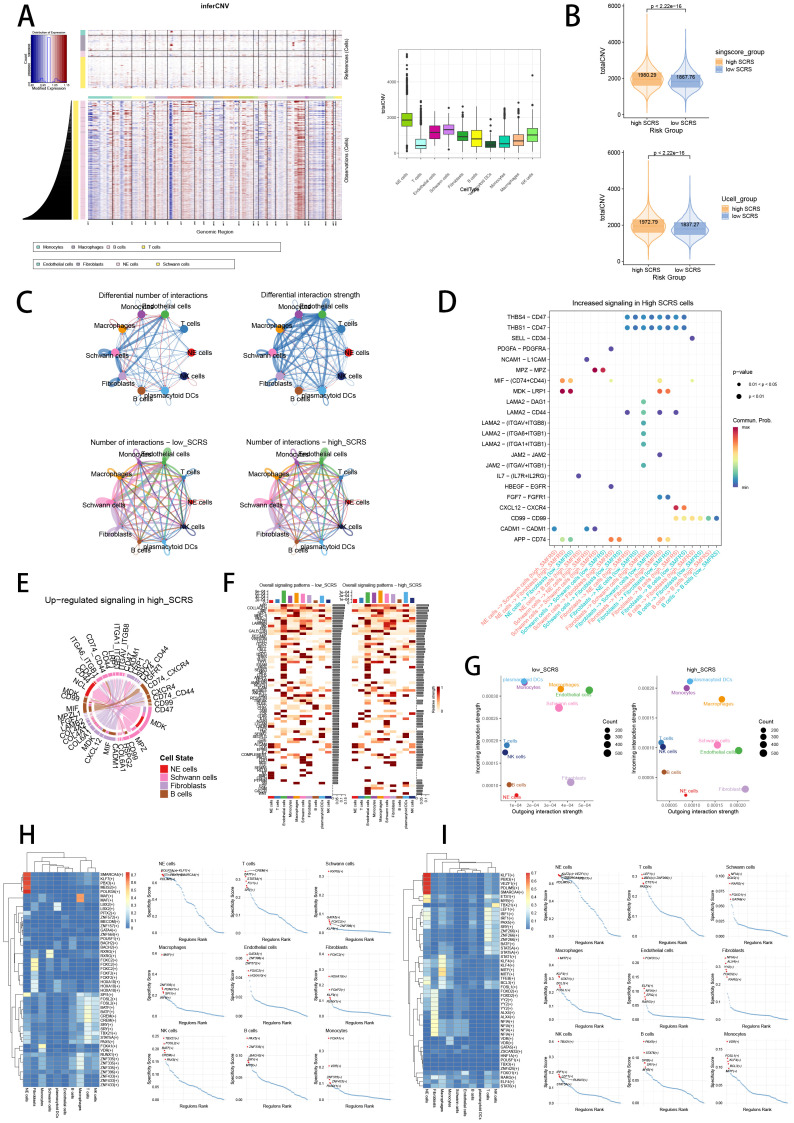
The landscape of CNV, cell-cell communication, transcriptional regulons in GSE137804 scRNA-seq cohort. **(A, B)** Significant differences of CNVs in NE cells, fibroblasts, Schwann cells and endothelial cells in high-SCRS cells and low-SCRS cells. **(C)** Circle diagrams showed the interaction strength and numbers between each cell type in high-SCRS cells and low-SCRS cells. **(D)** Bubble chart showed differences in communication signals between high-SCRS cells and low-SCRS cells. Bubble size represents P value generated by the permutation test, and the color represents the possibility of interactions. **(E)** Chord chart showed overexpressed ligand–receptor interactions in high-SCRS cells. **(F)** Heatmap showed the efferent or afferent contributions of all signals to different cell types in low-SCRS cells (left) and high-SCRS cells (right). **(G)** Dot plot showed dominant senders and receivers in low-SCRS cells (left) and high-SCRS cells (right). The X and Y axes are the total outgoing or incoming communication probabilities associated with each cell group, respectively. The size of the dots is positively related to the number of inferred links (both outgoing and incoming) associated with each cell block. The colors of the dots represent different cell groups. **(H)** SCENIC analysis indicated significant regulons in high-SCRS cells and TF rank plots of each cell type. **(I)** SCENIC analysis indicated significant regulons in low-SCRS cells and TF rank plots of each cell type.

Validating the power of SCRS in single cell level, we used inferCNV analysis to explore the clone structures of four cell types mentioned above, which indicated that NE cells with chromosome 17q gain were probably malignant cells, while high-SCRS cells owned more CNVs (**Figures 13A, B**). In cell chat analysis, we utilized “circle plot” to display the communication frequencies and communication strengths between every cell type, as well as the integrative cell chatting networks in high-SCRS cells and low-SCRS cells (**Figure 13C**). We visualized the cell chat landscapes in high-SCRS cells, and demonstrated over-expressed ligand-receptor pairs and communication profiles between B cells, myeloid cells, Schwann cells and fibroblasts in high-SCRS cells (**Figures 13D, E**). Distinct cell types would generate dissimilar contributive cues affecting the total, inbound and outbound signals among high-SCRS and low-SCRS cells, with macrophages, monocytes, Schwann cells, fibroblast, and endothelial cells noting exceptional significance (**Figures 13F, G**; **Supplementary Figure S3G**). The cellular communication between CAF subtypes holds significant biological importance, we then explore the communication network of CAF subpopulations in NB and pan-cancer landscape (**Supplementary Figure S3H**). We calculated the correlations between the hub gene of senes CAFs and cell-cell communication strength, finding a significant association between JAK1 and communication strength of CAF subtypes in NB, which offered a research value of JAK1 in modulating cellular communication (**Supplementary Figure S3I**). Meanwhile, we assessed the association among regulon (TFs and target genes) activities and cell types in high-SCRS and low-SCRS cells via SCENIC, revealing that regulons of SMARCA4 and PBX3 were more active in high SCRS cells (**Figure 13H**), and regulons of VEZF1 and PDLIM5 were more active in low SCRS cells (**Figure 13I**).

### Pan-cancer landscape and spatial transcriptomics analysis of hub gene

**Figure 14:**
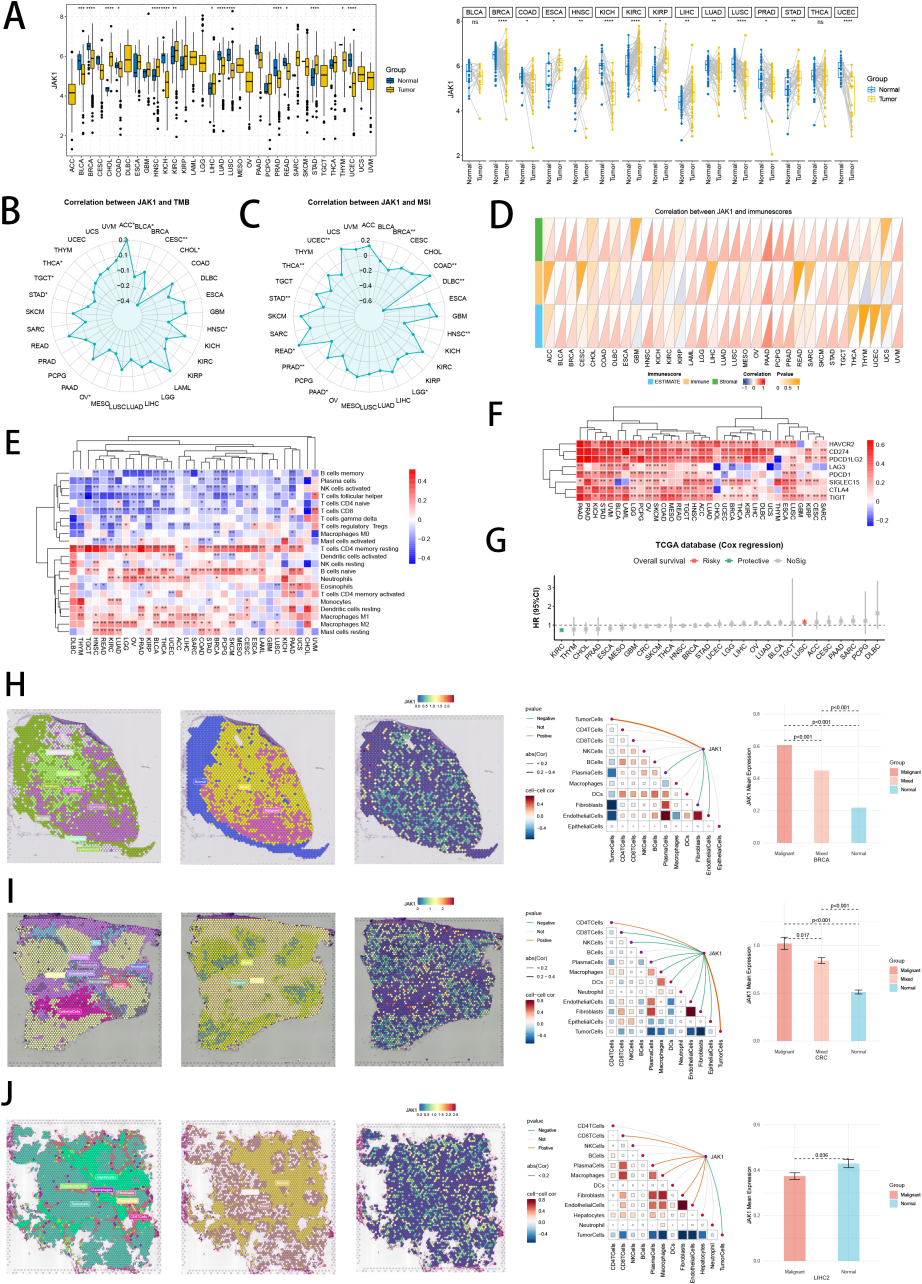
Pan-cancer analysis and spatial transcriptomics analysis of hub gene JAK1. **(A)** Differential expressions of JAK1 in tumor and normal samples across 33 tumor types. ns: not significant; *p < 0.05; **p < 0.01; ***p < 0.001; ****p < 0.0001. **(B, C)** Correlation analysis between expressions of JAK1 and TMB/MSI scores. **(D, E)** Correlation analysis between expressions of JAK1 and immune cell proportions/immune scores calculated by ESTIMATE and CIBERSORT. **(F)** Correlation analysis between expressions of JAK1 and immune checkpoint genes. *p < 0.05; **p < 0.01; ***p < 0.001; ****p < 0.0001. **(G)** Cox regression analysis of JAK1 in multiple tumor types. **(H-J)** Pan-cancer spatial transcriptomics analysis of JAK1 in BRCA, CRC and LIHC. Left one: Each dot is a microregion of spatial transcriptome sequencing, and a different color represents a different cell type. Left two: Spatial feature plots of malignant, mixed and normal areas via “Cottrazm” analysis. Left three: Spatial feature plots of gene expression of JAK1. Left four: Spearman correlation analysis calculated the correlations between one cell count and another cell count, and between cell count and gene expression in all spots. Left five: The horizontal coordinate is the different microregion types, and the vertical coordinate is the average expression of JAK1. Wilcoxon Rank Sum Tests assessed the significance of statistical differences.

Interestingly, we revealed that hub gene JAK1, with favorable prognosis value in NB, was abundant in oncological research value, which has been proved as oncogene in breast cancer and non-small cell lung cancer (79). Therefore, we conducted pan-cancer analysis to explore the heterogeneity of JAK1expression in tumor and normal samples in 33 cancer types (**Figure 14A**). Besides, the links between JAK1 and TMB, MSI, immune cells, and immunological scores underscored importance of JAK1 in TME, immune cell invasions, and immune checkpoints (**Figures 14B–F**). The protective prognostic value of JAK1 was seen in KIRC, similar to NB. The risky prognostic value of JAK1was seen in LUSC (**Figure 14G**). Furthermore, we conducted pan-cancer spatial transcriptomics analysis to comprehensively explore the expressions of JAK1 in malignant cells and malignant spots. In tumor types of BRCA, CRC, HNSC, CESC, GIST, KIRC, LUSC and OVCA (**Figures 14H, I**; **Supplementary Figures S4A–F**), hub gene JAK1 solidly exhibited positive correlations with the abundance of malignant cells across eight cancer types, while expressing higher in malignant areas than in normal areas in multiple spatial transcriptomics slides, indicating the underlying oncogenic role of JAK1. Interestingly, hub gene JAK1 abnormally exhibited negative correlations with the abundance of malignant cells in LIHC spatial transcriptomics slide, while expressing lower in malignant areas than in normal areas (**Figure 14J**). This result suggests a unique functional role for JAK1 in this particular cancer type, which highlights the complexity of cancer biology and the importance of context in gene function.

### Immunohistochemistry of hub gene

**Figure 15:**
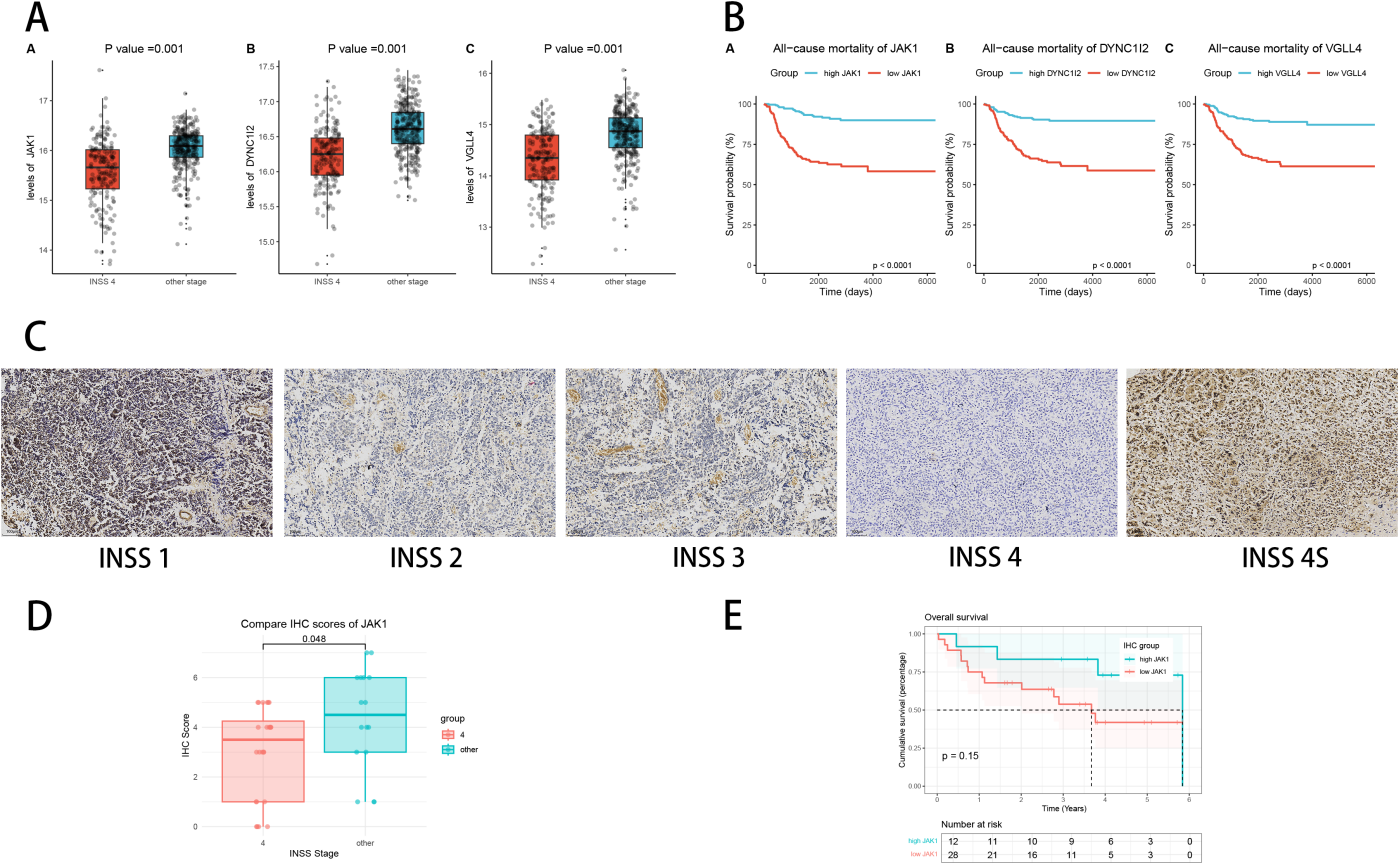
Experimental validation of hub gene JAK1 by Immunohistochemistry. **(A)** Different expressions of three hub genes (JAK1, DYNC1I2 and VGLL4) in INSS stage 4 tumors and other INSS stages tumors in GSE49710 cohort. **(B)** Protective prognosis value of three hub genes (JAK1, DYNC1I2 and VGLL4) illustrated by K-M curves in GSE49710 cohort. **(C)** Representative IHC staining pictures of INSS 1, 2, 3, 4 and 4S tumor samples. **(D)** Protein levels of JAK1 in INSS stage 4 tumors and other INSS stages tumors via IHC scores. **(E)** K-M curves of high-JAK1 group and low-JAK1 group.

Our bioinformatics analysis has revealed that three hub genes, which were both diagnostic model genes as well as prognostic model genes in SCRS, were expressed significantly lower in stage 4 NB (**Figure 15A**) and were protective prognosis genes (**Figure 15B**) in GSE49710 cohort. To validate the elevated protein levels of JAK1 in other stages NB tissues compared to stage 4 NB tissues, as well as its protective prognosis value, we conducted IHC staining to corroborate our bioinformatics findings (**Figure 15C**). Our scoring results showed that protein levels of JAK1 was significantly higher in other stages NB tissues than stage 4 NB tissues (**Figure 15D**). Subsequently, we categorized 40 NB patients into high and low expression groups based on their median IHC scores of JAK1. Survival analysis indicated that patients with high JAK1 expression had better OS than those with low JAK1 levels, without significance (p = 0.15, **Figure 15E**).

## Discussion

Neuroblastoma (NB), primarily diagnosed in children below the age of five, holds significant responsibility for around 15% of tumor-associated fatalities in pediatric settings (80). The diagnosis and therapeutic strategies are complicated due to the unique clinical manifestations and molecular characteristics of NB (81). In severe disease with advanced stages, complete surgical tumor removal becomes increasingly difficult. This is largely because the tumor engulfs and destructs the neurovascular structure subsequent to expansive growth. Moreover, advanced stages are typically associated with potentially severe conditions like recurrent relapse, remote metastasis and drug resistance. Consequently, this results in notably unfavorable outcomes for those grappling with NB (82). This challenging context has called the urgent need to develop more effective individualized treatment regimens, find new therapeutic targets, and reduce long-term drug side effects for NB patients.

The complex biological background and diverse clinic characteristics of NB pose huge challenges for doctors and clinicians. Advances in high-throughput sequencing methodologies has prompted the unveiling of unique prognostic and diagnostic markers, thus enabling more precise estimations of patient outcomes and personalized treatment approaches. Previous studies on senescence have focused on mechanisms, functions and treatment innovations, our research aims to explore the biological and clinical roles of senescence related CAFs (senes CAFs) in NB. We have systematically gathered a detailed list of senescence-related genes for thorough investigation. Through a comprehensive bioinformatics analysis combining both scRNA and bulk-seq, we successfully identified a distinct CAF subtype namely senes CAF, as well as numerous critical marker genes of senes CAF. Subsequently, we were able to construct senes CAF centric predictive models based on marker genes with great prognosis value, referred to SCRS, employing integrated machine-learning techniques. SCRS demonstrated its proficiency for accurately diagnosing stage 4 NB and projecting the prognosis for NB patients, showcasing an impressive ability to foresee immunotherapy responses and to categorize patients into high or low risk across various factors, such as immune microenvironment, mutational landscapes, chemotherapy responsiveness, and single-cell resolution. Moreover, we distinguished two distinct NB subtypes via consensus clustering, each characterized by valuable senes CAF marker genes, which shed light on molecular landscape of senes CAF in NB.

Machine earning (ML) serves as an essential tool in our research, utilizing sophisticated algorithms to automatically manage expansive and varied datasets. Its optimal operation lies in predictive tasks where it identifies significant patterns (83). We relied on an integrated ML framework to establish a consensus SCRS using the expression patterns of senes CAF marker genes, aiming at diagnosing stage 4 NB and forecasting NB prognosis. A total of 101 prognosis algorithms and 113 diagnosis algorithms were implemented in the train set according to LOOCV framework. Further corroborations in another four datasets disclosed that the most effective prognosis algorithm was SuperPC in feature selection and model construction, and the most reliable diagnosis algorithm was RF in feature selection and model development. The robustness of this unified method lies within its capacity to assemble multiple ML algorithms, and that results into creating models with persistent diagnosis or prognosis abilities. This method simplifies the model for functional and translational use by reducing the dimensionality of numerous variables. The performance of SCRS was validated by confusion matrix, time-dependent ROC curves, AUC values, calibration curves, and DCA curves, all of which accentuate its supremacy over other clinic factors. In addition, a meta-analysis of C-index indicated that prognostic SCRS retained highest precision and robustness across external validation datasets, indicating its significant prospects in clinic use and helping decision-making.

With the risk-stratify ability of prognostic SCRS, it allowed us to discover the biological discrepancies and genetic mechanisms between two risk groups. Notable biological diversity was observed between two risk groups in terms of immune microenvironment, responsiveness to immunotherapy, somatic mutations, and chemotherapy sensitivity. We conducted a functional enrichment analysis with DEGs between two risk groups, which revealed that the key differences were primarily concentrated in areas like spermatogenesis, oocyte meiosis, and cell cycle. NB could potentially be the result of aberrant development of neural crest stem cells, which is linked to mis-regulation of differentiation, morphogenesis, and cell cycle. The migratory pathways of neural crest stem cells correspond to tumoral locations of INSS stage 4S and 4 NB, encompassing adrenal gland, liver, and bone marrow. It’s noteworthy that most malignant tumors have been discovered to retain stem cells or precursor-like cells with stem cell features, which are linked to impact of genetic and epigenetic alterations on differentiating and mature cells (84). We perform function enrichment analysis to reveal a possibility of SCRS model genes being implicated in development of NB tumor cells, which could potentially affect the signals and pathways associated with differentiation and maturation.

Immunotherapy extends survival prospects for patients with malignant tumors, providing potential encouragement for those afflicted with this debilitating disease. Our analysis of the interactions between SCRS and TME exposed a negative association between SCRS risk scores and the majority of immune cells and immunologically regulatory genes. Function enrichment analysis pointed towards an elevation of immunoregulatory function levels in low-risk group. Hence, low-risk group often display symptoms consistent with “immune activity” or “hot tumor”, which is typified by increased infiltration of various immunocytes. Significantly, past research underlines that increased presence of immune cells in tumor microenvironment often correlates with better prognoses for NB, suggesting promising capacity to hamper cancer progression. This conjecture was also verified in four immunotherapy bulk-seq datasets, supporting the viewpoint that SCRS was a promising prediction signature for immunotherapy effectiveness.

Subsequently, we performed single cell analysis to thoroughly explore the underlying biological functions of SCRS model genes. Utilizing six distinct single-cell scoring algorithms, we were able to vividly illustrate the expression landscapes of SCRS model genes at individual cell levels, demonstrating substantial infiltration in malignant NE cells. We utilized bulk-seq datasets to categorize samples into two markedly different phenotypes, namely high-SCRS and low-SCRS. The Scissor algorithm was then implemented to map concerned phenotypes into the scRNA data, aiming at pinpointing the cells most tightly related to high or low SCRS profile. Consequently, the low and high SCRS statuses were respectively represented by the Scissor- cells and Scissor+ cells, showing significant distinctions in terms of senescence. We further initiated pseudo-time analysis and employed the inferCNV method individually in high-SCRS and low-SCRS cells, which elicited remarkable disparities in cell maturation and mutational landscapes across two SCRS categories. We found that cells displaying elevated SCRS scores were predominantly immature and malignant cells, supporting the aptitude of SCRS for risk stratification. Additionally, biological differences were highlighted in the cellular interaction and transcriptional regulon networks in two SCRS groups, contributing to a more comprehensive comprehension of cancer microenvironment and cancer heterogeneity, based on cell chat analysis and SCENIC algorithm.

Janus kinase 1 (JAK1) is a critical player in various cellular processes, including inflammation, immune response, and oncogenesis, due to its central role in the JAK-STAT signaling pathway (85). Subsequently, based on spatial transcriptomics analysis, we found expression of hub gene JAK1 was positively correlated to the proportions of malignant cells in spatial transcriptomics spots in most cancer types excluding LIHC, indicating its malignant phenotypes and heterogeneity in pan-cancer levels. Interestingly, we utilized “Cottrazm” to combine spatial transcriptome data with HE staining images and single cell transcriptome data, mapping the cancer boundary region linking malignant and non-malignant regions in cancer tissue accurately. Unlike the protective role of JAK1 in NB, we revealed that JAK1 expressed significantly higher in malignant area than in mix area or normal area, indicating its huge abundance in tumor cells at spatial dimension in most adult tumors. In LIHC, JAK1 might interact with other signaling pathways in liver cells that result in tumor suppression. For instance, it could activate STAT proteins that induce the expression of genes responsible for cell cycle arrest or apoptosis, thereby inhibiting cancer cell growth and survival (86).

Our SCRS can be conveniently replicated using PCR detective techniques, which is feasible for broader clinic application and utilization. Sequencing the genome or transcriptome of NB patient samples obtained from biopsies or surgeries could serve as a routine diagnostic tool to inform treatment strategies. With this data integrated into SCRS, clinicians could accurately stage INSS 4 NB, assess prognosis, and design tailored treatments for patients facing distant metastasis and challenging outcomes. Nevertheless, it’s important to note some limitations within our study. Initially, our study was carried out retrospectively, with sequencing data and relevant clinical info gathered from public archives, which needs a large-scale, multi-center prospective validation. And the lack of details therapy procedures, metastasis organs, and recurrence data could potentially influence our findings. Secondly, the characteristics of JAK1 in NB have not been conclusively identified, therefore further research involving additional tumor samples, and more *in vitro* or *in vivo* experimental investigations are required to explore their biological roles within NB. At last, our present methodology of model development, which depends entirely on transcriptome sequencing, could gain significantly from integrative analysis of multi-omics and multi-modal data. This well-rounded integration analysis enables a more thorough understanding of molecular mechanism and physiological process, refining the reliability and precision of the prediction models. The inclusion of multi-omics and multi-modal data introduces a bevy of variables to the analysis, which is imperative for more intricate artificial intelligence models. Hence, deep learning, a specialized field within machine learning, possesses the unique ability to independently identify crucial classification features. This option isn’t readily available with conventional machine learning techniques, which require manual selection and input of such features. Consequently, the adoption and deployment of novel deep learning algorithms, alongside the insightful benefits afforded by multi-omics and multi-modal data integration, denote a powerful strategy for progressing personalized medicine for NB patients.

## Conclusion

For the first time, we have been successful in developing senes CAFs related signatures to accurately diagnose INSS stage 4 NB and predict prognosis in NB, thanks to a wealth of machine learning algorithms. After multiple validations in model performance, immune microenvironment, mutational landscapes, immunotherapy, chemotherapy, single cell resolution and spatial transcriptomics analysis, SCRS has demonstrated both stability and potency in outcome prediction, making it a remarkable prediction model in NB. Furthermore, we revealed hub gene JAK1 with huge impact in SCRS, which showed heterogeneous prognosis value in pan-cancer landscapes, suggesting potential research opportunities associated with senes CAFs.

## Data Availability

The original contributions presented in the study are included in the article/**Supplementary Material**. Further inquiries can be directed to the corresponding authors.

## Supplementary material

Supplementary Figure 1
**(A)** The workflow chart of our study. **(B)** Visualizing the distribution of various cancer types by UMAP plot in pan-cancer landscape. **(C)** Quality control of CAF subtype in pan-cancer scRNA-seq cohort. **(D)** Harmony algorithm reduced the batch effects between each cancer type. **(E)** Boxplot showing the local inverse Simpson’s Index (LISI) of fibroblasts before and after batch correction. **(F)** Five scRNA scoring algorithms visualized the senescence enriched scores of each CAF subpopulation. **(G)** KEGG enrichment analysis showed each CAF subtype’s top 3 functional terms. **(H)** UMAP plots showing the co-embedding results of scRNA-seq and ST data using CellTrek.

Supplementary Figure 2
**(A)** Quality control of each major cell type in GSE137804 scRNA-seq cohort. **(B)** Harmony algorithm reduced the batch effects of each sample in GSE137804 scRNA-seq cohort. **(C)** Quality control of each subtype in CAF subpopulations. **(D)** Harmony algorithm reduced the batch effects of each sample in CAF subpopulations. **(E)** UMAP plot showed the expression profiles of marker genes. **(F)** Six scRNA scoring algorithms visualized the senescence enriched scores of each CAF subpopulation. **(G)** Visualizing the distribution of major cell type and CAF subpopulations by UMAP plot in other NB datasets. **(H)** scRNA scoring algorithm of AUCell ranking visualized the senescence enriched scores of each CAF subpopulation. **(I)** Scatter plot showing the regulon specificity scores (RSSs) in each CAF subtype via SCENIC analysis. The top 5 regulons are highlighted.

Supplementary Figure 3
**(A)** PCA plots visualized the well corrections after batch removement in five bulk-seq cohorts. **(B)** Acquisition of diagnostic and prognostic senescence-related genes via intersection of genes with diagnosis or prognosis value in four bulk-seq cohorts. **(C)** The flowchart to schematically explain the algorithmic pipeline of machine learning algorithm integration. **(D)** The performance of five ML models in terms of Area under precision-recall curve (PRAUC), accuracy, sensitivity, specificity, precision, cross-entropy, Brier scores, balanced accuracy and F1 Score in five bulk-seq cohorts. **(E)** The precision-recall curves (PRC) in two bulk-seq cohort. **(F)** Logarithmic loss, recall and decision calibration of top 5 prognostic machine learning models in five bulk-seq cohorts. **(G)** The combined heatmap shows the results after NicheNet analysis of high-SCRS cells and low-SCRS cells. **(H)** Circle diagrams showed the interaction strength and numbers between each CAF subtype in high-SCRS cells and low-SCRS cells in NB and pan-cancer landscape. **(I)** The spearman correlations between the hub gene of senes CAFs and cell-cell communication strengths in NB and pan-cancer landscape.

Supplementary Figure 4 Pan-cancer spatial transcriptomics analysis of JAK1 in HNSC **(A)**, CESC **(B)**, GIST **(C)**, KIRC **(D)**, LUSC **(E)** and OVCA **(F)**. Left one: Each dot is a microregion of spatial transcriptome sequencing, and a different color represents a different cell type. Left two: Spatial feature plots of malignant, mixed and normal areas via “Cottrazm” analysis. Left three: Spatial feature plots of gene expression of JAK1. Left four: Spearman correlation analysis calculated the correlations between one cell count and another cell count, and between cell count and gene expression in all spots. Left five: The horizontal coordinate is the different microregion types, and the vertical coordinate is the average expression of JAK1. Wilcoxon Rank Sum Tests assessed the significance of statistical differences.

## References

[1] Tsubota S Kadomatsu K. Origin and initiation mechanisms of neuroblastoma. Cell Tissue Res. (2018) 372:211–21. doi: 10.1007/s00441-018-2796-z 29445860

[2] Kholodenko IV Kalinovsky DV Doronin II Deyev SM Kholodenko RV. Neuroblastoma origin and therapeutic targets for immunotherapy. J Immunol Res. (2018) 2018:7394268. doi: 10.1155/2018/7394268 30116755 PMC6079467

[3] Irwin MS Naranjo A Zhang FF Cohn SL London WB Gastier-Foster JM. Revised neuroblastoma risk classification system: A report from the children’s oncology group. J Clin Oncol. (2021) 39:3229–41. doi: 10.1200/JCO.21.00278 PMC8500606 34319759

[4] Brodeur GM Pritchard J Berthold F Carlsen NL Castel V Castelberry RP. Revisions of the international criteria for neuroblastoma diagnosis, staging, and response to treatment. J Clin Oncol. (1993) 11:1466–77. doi: 10.1200/JCO.1993.11.8.1466 8336186

[5] Dong R Yang R Zhan Y Lai HD Ye CJ Yao XY. Single-cell characterization of Malignant phenotypes and developmental trajectories of adrenal neuroblastoma. Cancer Cell. (2020) 38:716–733.e6. doi: 10.1016/j.ccell.2020.08.014 32946775

[6] Xu M Zhang T Xia R Wei Y Wei X. Targeting the tumor stroma for cancer therapy. Mol Cancer. (2022) 21:208. doi: 10.1186/s12943-022-01670-1 36324128 PMC9628074

[7] Chhabra Y Weeraratna AT. Fibroblasts in cancer: Unity in heterogeneity. Cell. (2023) 186:1580–609. doi: 10.1016/j.cell.2023.03.016 PMC11422789 37059066

[8] Faget DV Ren Q Stewart SA. Unmasking senescence: context-dependent effects of SASP in cancer. Nat Rev Cancer. (2019) 19:439–53. doi: 10.1038/s41568-019-0156-2 31235879

[9] Frey N Venturelli S Zender L Bitzer M. Cellular senescence in gastrointestinal diseases: from pathogenesis to therapeutics. Nat Rev Gastroenterol Hepatol. (2018) 15:81–95. doi: 10.1038/nrgastro.2017.146 29184185

[10] Hernandez-Segura A de Jong TV Melov S Guryev V Campisi J Demaria M. Unmasking transcriptional heterogeneity in senescent cells. Curr Biol. (2017) 27:2652–2660.e4. doi: 10.1016/j.cub.2017.07.033 28844647 PMC5788810

[11] Krizhanovsky V Yon M Dickins RA Hearn S Simon J Miething C. Senescence of activated stellate cells limits liver fibrosis. Cell. (2008) 134:657–67. doi: 10.1016/j.cell.2008.06.049 PMC3073300 18724938

[12] Ruhland MK Coussens LM Stewart SA. Senescence and cancer: An evolving inflammatory paradox. Biochim Biophys Acta. (2016) 1865:14–22. doi: 10.1016/j.bbcan.2015.10.001 26453912 PMC4733607

[13] Liu Z Liu L Weng S Guo C Dang Q Xu H. Machine learning-based integration develops an immune-derived lncRNA signature for improving outcomes in colorectal cancer. Nat Commun. (2022) 13:816. doi: 10.1038/s41467-022-28421-6 35145098 PMC8831564

[14] Leek JT Johnson WE Parker HS Jaffe AE Storey JD. The sva package for removing batch effects and other unwanted variation in high-throughput experiments. Bioinformatics. (2012) 28:882–3. doi: 10.1093/bioinformatics/bts034 PMC3307112 22257669

[15] Satija R Farrell JA Gennert D Schier AF Regev A. Spatial reconstruction of single-cell gene expression data. Nat Biotechnol. (2015) 33:495–502. doi: 10.1038/nbt.3192 25867923 PMC4430369

[16] McGinnis CS Murrow LM Gartner ZJ. DoubletFinder: doublet detection in single-cell RNA sequencing data using artificial nearest neighbors. Cell Syst. (2019) 8:329–337.e4. doi: 10.1016/j.cels.2019.03.003 30954475 PMC6853612

[17] Korsunsky I Millard N Fan J Slowikowski K Zhang F Wei K. Fast, sensitive and accurate integration of single-cell data with Harmony. Nat Methods. (2019) 16:1289–96. doi: 10.1038/s41592-019-0619-0 PMC6884693 31740819

[18] Butler A Hoffman P Smibert P Papalexi E Satija R. Integrating single-cell transcriptomic data across different conditions, technologies, and species. Nat Biotechnol. (2018) 36:411–20. doi: 10.1038/nbt.4096 PMC6700744 29608179

[19] Ma C Yang C Peng A Sun T Ji X Mi J. Pan-cancer spatially resolved single-cell analysis reveals the crosstalk between cancer-associated fibroblasts and tumor microenvironment. Mol Cancer. (2023) 22:170. doi: 10.1186/s12943-023-01876-x 37833788 PMC10571470

[20] Cords L Tietscher S Anzeneder T Langwieder C Rees M de Souza N. Cancer-associated fibroblast classification in single-cell and spatial proteomics data. Nat Commun. (2023) 14:4294. doi: 10.1038/s41467-023-39762-1 37463917 PMC10354071

[21] Andreatta M Carmona SJ. UCell: Robust and scalable single-cell gene signature scoring. Comput Struct Biotechnol J. (2021) 19:3796–8. doi: 10.1016/j.csbj.2021.06.043 PMC8271111 34285779

[22] Foroutan M Bhuva DD Lyu R Horan K Cursons J Davis MJ. Single sample scoring of molecular phenotypes. BMC Bioinf. (2018) 19:404. doi: 10.1186/s12859-018-2435-4 PMC6219008 30400809

[23] Hänzelmann S Castelo R Guinney J. GSVA: gene set variation analysis for microarray and RNA-seq data. BMC Bioinf. (2013) 14:7. doi: 10.1186/1471-2105-14-7 PMC3618321 23323831

[24] Zheng L Qin S Si W Wang A Xing B Gao R. Pan-cancer single-cell landscape of tumor-infiltrating T cells. Science. (2021) 374:abe6474. doi: 10.1126/science.abe6474 34914499

[25] Gulati GS Sikandar SS Wesche DJ Manjunath A Bharadwaj A Berger MJ. Single-cell transcriptional diversity is a hallmark of developmental potential. Science. (2020) 367:405–11. doi: 10.1126/science.aax0249 PMC7694873 31974247

[26] Street K Risso D Fletcher RB Das D Ngai J Yosef N. Slingshot: cell lineage and pseudotime inference for single-cell transcriptomics. BMC Genomics. (2018) 19:477. doi: 10.1186/s12864-018-4772-0 29914354 PMC6007078

[27] Cao J Spielmann M Qiu X Huang X Ibrahim DM Hill AJ. The single-cell transcriptional landscape of mammalian organogenesis. Nature. (2019) 566:496–502. doi: 10.1038/s41586-019-0969-x 30787437 PMC6434952

[28] Cable DM Murray E Zou LS Goeva A Macosko EZ Chen F. Robust decomposition of cell type mixtures in spatial transcriptomics. Nat Biotechnol. (2022) 40:517–26. doi: 10.1038/s41587-021-00830-w PMC8606190 33603203

[29] Han Y Wang Y Dong X Sun D Liu Z Yue J. TISCH2: expanded datasets and new tools for single-cell transcriptome analyses of the tumor microenvironment. Nucleic Acids Res. (2023) 51:D1425–d1431. doi: 10.1093/nar/gkac959 36321662 PMC9825603

[30] Xun Z Ding X Zhang Y Zhang B Lai S Zou D. Reconstruction of the tumor spatial microenvironment along the Malignant-boundary-nonmalignant axis. Nat Commun. (2023) 14:933. doi: 10.1038/s41467-023-36560-7 36806082 PMC9941488

[31] Wei R He S Bai S Sei E Hu M Thompson A. Spatial charting of single-cell transcriptomes in tissues. Nat Biotechnol. (2022) 40:1190–9. doi: 10.1038/s41587-022-01233-1 PMC9673606 35314812

[32] Lang M Binder M Richter J Schratz P Pfisterer F Coors S. mlr3: A modern object-oriented machine learning framework in R. J Open Source Software. (2019) 4:1903. doi: 10.21105/joss.01903

[33] Sonabend R Király FJ Bender A Bischl B Lang M. mlr3proba: an R package for machine learning in survival analysis. Bioinformatics. (2021) 37:2789–91. doi: 10.1093/bioinformatics/btab039 PMC8428574 33523131

[34] Wilkerson MD Hayes DN. ConsensusClusterPlus: a class discovery tool with confidence assessments and item tracking. Bioinformatics. (2010) 26:1572–3. doi: 10.1093/bioinformatics/btq170 PMC2881355 20427518

[35] Yu G Wang LG Han Y He QY. clusterProfiler: an R package for comparing biological themes among gene clusters. Omics. (2012) 16:284–7. doi: 10.1089/omi.2011.0118 PMC3339379 22455463

[36] Subramanian A Tamayo P Mootha VK Mukherjee S Ebert BL Gillette MA. Gene set enrichment analysis: a knowledge-based approach for interpreting genome-wide expression profiles. Proc Natl Acad Sci USA. (2005) 102:15545–50. doi: 10.1073/pnas.0506580102 PMC1239896 16199517

[37] Zeng D Ye Z Shen R Yu G Wu J Xiong Y. IOBR: multi-omics immuno-oncology biological research to decode tumor microenvironment and signatures. Front Immunol. (2021) 12:687975. doi: 10.3389/fimmu.2021.687975 34276676 PMC8283787

[38] Newman AM Liu CL Green MR Gentles AJ Feng W Xu Y. Robust enumeration of cell subsets from tissue expression profiles. Nat Methods. (2015) 12:453–7. doi: 10.1038/nmeth.3337 PMC4739640 25822800

[39] Yoshihara K Shahmoradgoli M Martínez E Vegesna R Kim H Torres-Garcia W. Inferring tumour purity and stromal and immune cell admixture from expression data. Nat Commun. (2013) 4:2612. doi: 10.1038/ncomms3612 24113773 PMC3826632

[40] Finotello F Mayer C Plattner C Laschober G Rieder D Hackl H. Molecular and pharmacological modulators of the tumor immune contexture revealed by deconvolution of RNA-seq data. Genome Med. (2019) 11:34. doi: 10.1186/s13073-019-0638-6 31126321 PMC6534875

[41] Li B Severson E Pignon JC Zhao H Li T Novak J. Comprehensive analyses of tumor immunity: implications for cancer immunotherapy. Genome Biol. (2016) 17:174. doi: 10.1186/s13059-016-1028-7 27549193 PMC4993001

[42] Charoentong P Finotello F Angelova M Mayer C Efremova M Rieder D. Pan-cancer immunogenomic analyses reveal genotype-immunophenotype relationships and predictors of response to checkpoint blockade. Cell Rep. (2017) 18:248–62. doi: 10.1016/j.celrep.2016.12.019 28052254

[43] Becht E Giraldo NA Lacroix L Buttard B Elarouci N Petitprez F. Estimating the population abundance of tissue-infiltrating immune and stromal cell populations using gene expression. Genome Biol. (2016) 17:218. doi: 10.1186/s13059-016-1070-5 27765066 PMC5073889

[44] Aran D Hu Z Butte AJ. xCell: digitally portraying the tissue cellular heterogeneity landscape. Genome Biol. (2017) 18:220. doi: 10.1186/s13059-017-1349-1 29141660 PMC5688663

[45] Racle J de Jonge K Baumgaertner P Speiser DE Gfeller D. Simultaneous enumeration of cancer and immune cell types from bulk tumor gene expression data. Elife. (2017) 13:6. doi: 10.7554/eLife.26476 PMC5718706 29130882

[46] Jia Q Wu W Wang Y Alexander PB Sun C Gong Z. Local mutational diversity drives intratumoral immune heterogeneity in non-small cell lung cancer. Nat Commun. (2018) 9:5361. doi: 10.1038/s41467-018-07767-w 30560866 PMC6299138

[47] Barbie DA Tamayo P Boehm JS Kim SY Moody SE Dunn IF. Systematic RNA interference reveals that oncogenic KRAS-driven cancers require TBK1. Nature. (2009) 462:108–12. doi: 10.1038/nature08460 PMC2783335 19847166

[48] Xu L Deng C Pang B Zhang X Liu W Liao G. TIP: A web server for resolving tumor immunophenotype profiling. Cancer Res. (2018) 78:6575–80. doi: 10.1158/0008-5472.CAN-18-0689 30154154

[49] Thorsson V Gibbs DL Brown SD Wolf D Bortone DS Ou Yang TH. The immune landscape of cancer. Immunity. (2018) 48:812–830.e14. doi: 10.1016/j.immuni.2018.03.023 29628290 PMC5982584

[50] Mayakonda A Lin DC Assenov Y Plass C Koeffler HP. Maftools: efficient and comprehensive analysis of somatic variants in cancer. Genome Res. (2018) 28:1747–56. doi: 10.1101/gr.239244.118 PMC6211645 30341162

[51] Mermel CH Schumacher SE Hill B Meyerson ML Beroukhim R Getz G. GISTIC2.0 facilitates sensitive and confident localization of the targets of focal somatic copy-number alteration in human cancers. Genome Biol. (2011) 12:R41. doi: 10.1186/gb-2011-12-4-r41. 21527027 PMC3218867

[52] Zhang H Meltzer P Davis S. RCircos: an R package for Circos 2D track plots. BMC Bioinf. (2013) 14:244. doi: 10.1186/1471-2105-14-244 PMC3765848 23937229

[53] Jiang P Gu S Pan D Fu J Sahu A Hu X. Signatures of T cell dysfunction and exclusion predict cancer immunotherapy response. Nat Med. (2018) 24:1550–8. doi: 10.1038/s41591-018-0136-1 PMC6487502 30127393

[54] Roh W Chen PL Reuben A Spencer CN Prieto PA Miller JP. Integrated molecular analysis of tumor biopsies on sequential CTLA-4 and PD-1 blockade reveals markers of response and resistance. Sci Transl Med. (2017) 9(379). doi: 10.1126/scitranslmed.aah3560 PMC5819607 28251903

[55] Yang C Huang X Li Y Chen J Lv Y Dai S. Prognosis and personalized treatment prediction in TP53-mutant hepatocellular carcinoma: an in silico strategy towards precision oncology. Brief Bioinform. (2021) 22(3). doi: 10.1093/bib/bbaa164 32789496

[56] Sun D Guan X Moran AE Wu LY Qian DZ Schedin P. Identifying phenotype-associated subpopulations by integrating bulk and single-cell sequencing data. Nat Biotechnol. (2022) 40:527–38. doi: 10.1038/s41587-021-01091-3 PMC9010342 34764492

[57] Qiu X Hill A Packer J Lin D Ma YA Trapnell C. Single-cell mRNA quantification and differential analysis with Census. Nat Methods. (2017) 14:309–15. doi: 10.1038/nmeth.4150 PMC5330805 28114287

[58] Tirosh I Venteicher AS Hebert C Escalante LE Patel AP Yizhak K. Single-cell RNA-seq supports a developmental hierarchy in human oligodendroglioma. Nature. (2016) 539:309–13. doi: 10.1038/nature20123 PMC5465819 27806376

[59] Jin S Guerrero-Juarez CF Zhang L Chang I Ramos R Kuan CH. Inference and analysis of cell-cell communication using CellChat. Nat Commun. (2021) 12:1088. doi: 10.1038/s41467-021-21246-9 33597522 PMC7889871

[60] Browaeys R Saelens W Saeys Y. NicheNet: modeling intercellular communication by linking ligands to target genes. Nat Methods. (2020) 17:159–62. doi: 10.1038/s41592-019-0667-5 31819264

[61] Aibar S González-Blas CB Moerman T Huynh-Thu VA Imrichova H Hulselmans G. SCENIC: single-cell regulatory network inference and clustering. Nat Methods. (2017) 14:1083–6. doi: 10.1038/nmeth.4463 PMC5937676 28991892

[62] Liao C Wang X. TCGAplot: an R package for integrative pan-cancer analysis and visualization of TCGA multi-omics data. BMC Bioinf. (2023) 24:483. doi: 10.1186/s12859-023-05615-3 PMC10726608 38105215

[63] Chen Z Zhou L Liu L Hou Y Xiong M Yang Y. Single-cell RNA sequencing highlights the role of inflammatory cancer-associated fibroblasts in bladder urothelial carcinoma. Nat Commun. (2020) 11:5077. doi: 10.1038/s41467-020-18916-5 33033240 PMC7545162

[64] Liu C Zhang M Yan X Ni Y Gong Y Wang C. Single-cell dissection of cellular and molecular features underlying human cervical squamous cell carcinoma initiation and progression. Sci Adv. (2023) 9:eadd8977. doi: 10.1126/sciadv.add8977 36706185 PMC9882988

[65] Elyada E Bolisetty M Laise P Flynn WF Courtois ET Burkhart RA. Cross-species single-cell analysis of pancreatic ductal adenocarcinoma reveals antigen-presenting cancer-associated fibroblasts. Cancer Discovery. (2019) 9:1102–23. doi: 10.1158/2159-8290.CD-19-0094 PMC6727976 31197017

[66] Wang Y Liang Y Xu H Zhang X Mao T Cui J. Single-cell analysis of pancreatic ductal adenocarcinoma identifies a novel fibroblast subtype associated with poor prognosis but better immunotherapy response. Cell Discovery. (2021) 7:36. doi: 10.1038/s41421-021-00271-4 34035226 PMC8149399

[67] Galbo PM Jr. Zang X Zheng D. Molecular features of cancer-associated fibroblast subtypes and their implication on cancer pathogenesis, prognosis, and immunotherapy resistance. Clin Cancer Res. (2021) 27:2636–47. doi: 10.1158/1078-0432.CCR-20-4226 PMC8102353 33622705

[68] Chen B Chan WN Xie F Mui CW Liu X Cheung AHK. The molecular classification of cancer-associated fibroblasts on a pan-cancer single-cell transcriptional atlas. Clin Transl Med. (2023) 13:e1516. doi: 10.1002/ctm2.v13.12 38148640 PMC10751516

[69] Lavie D Ben-Shmuel A Erez N Scherz-Shouval R. Cancer-associated fibroblasts in the single-cell era. Nat Cancer. (2022) 3:793–807. doi: 10.1038/s43018-022-00411-z 35883004 PMC7613625

[70] Tang X Hou Y Yang G Wang X Tang S Du YE. Stromal miR-200s contribute to breast cancer cell invasion through CAF activation and ECM remodeling. Cell Death Differ. (2016) 23:132–45. doi: 10.1038/cdd.2015.78 PMC4815985 26068592

[71] Franceschi C Garagnani P Parini P Giuliani C Santoro A. Inflammaging: a new immune-metabolic viewpoint for age-related diseases. Nat Rev Endocrinol. (2018) 14:576–90. doi: 10.1038/s41574-018-0059-4 30046148

[72] Wu Y Yang S Ma J Chen Z Song G Rao D. Spatiotemporal immune landscape of colorectal cancer liver metastasis at single-cell level. Cancer Discovery. (2022) 12:134–53. doi: 10.1158/2159-8290.CD-21-0316 34417225

[73] Barkley D Moncada R Pour M Liberman DA Dryg I Werba G. Cancer cell states recur across tumor types and form specific interactions with the tumor microenvironment. Nat Genet. (2022) 54:1192–201. doi: 10.1038/s41588-022-01141-9 PMC9886402 35931863

[74] Sudmeier LJ Hoang KB Nduom EK Wieland A Neill SG Schniederjan MJ. Distinct phenotypic states and spatial distribution of CD8(+) T cell clonotypes in human brain metastases. Cell Rep Med. (2022) 3:100620. doi: 10.1016/j.xcrm.2022.100620 35584630 PMC9133402

[75] Liu Y Xun Z Ma K Liang S Li X Zhou S. Identification of a tumour immune barrier in the HCC microenvironment that determines the efficacy of immunotherapy. J Hepatol. (2023) 78:770–82. doi: 10.1016/j.jhep.2023.01.011 36708811

[76] Belle JI Sen D Baer JM Liu X Lander VE Ye J. Senescence defines a distinct subset of myofibroblasts that orchestrates immunosuppression in pancreatic cancer. Cancer Discovery. (2024) 14:1324–55. doi: 10.1158/2159-8290.CD-23-0428 PMC12155422 38683144

[77] Guo Y Zhou A Zhang Y Chen Y Chen Y Gao Y. Serum response factor activates peroxidasin transcription to block senescence of hepatic stellate cells. Life Sci. (2023) 328:121824. doi: 10.1016/j.lfs.2023.121824 37270170

[78] Kim J DeBerardinis RJ. Mechanisms and implications of metabolic heterogeneity in cancer. Cell Metab. (2019) 30:434–46. doi: 10.1016/j.cmet.2019.08.013 PMC6730674 31484055

[79] Shien K Papadimitrakopoulou VA Ruder D Behrens C Shen L Kalhor N. JAK1/STAT3 activation through a proinflammatory cytokine pathway leads to resistance to molecularly targeted therapy in non-small cell lung cancer. Mol Cancer Ther. (2017) 16:2234–45. doi: 10.1158/1535-7163.MCT-17-0148 PMC5628136 28729401

[80] Gurney JG Severson RK Davis S Robison LL. Incidence of cancer in children in the United States. Sex-, race-, and 1-year age-specific rates by histologic type. Cancer. (1995) 75:2186–95. doi: 10.1002/1097-0142(19950415)75:8<2186::AID-CNCR2820750825>3.0.CO;2-F 7697611

[81] Aygun N. Biological and genetic features of neuroblastoma and their clinical importance. Curr Pediatr Rev. (2018) 14:73–90. doi: 10.2174/1573396314666180129101627 29380702

[82] Bhatnagar SN Sarin YK. Neuroblastoma: a review of management and outcome. Indian J Pediatr. (2012) 79:787–92. doi: 10.1007/s12098-012-0748-2 22528697

[83] Goecks J Jalili V Heiser LM Gray JW. How machine learning will transform biomedicine. Cell. (2020) 181:92–101. doi: 10.1016/j.cell.2020.03.022 32243801 PMC7141410

[84] Ratner N Brodeur GM Dale RC. Schor NF. The “neuro” of neuroblastoma: Neuroblastoma as a neurodevelopmental disorder. Ann Neurol. (2016) 80:13–23. doi: 10.1002/ana.24659 27043043 PMC5419586

[85] Hosseini A Gharibi T Marofi F Javadian M Babaloo Z Baradaran B. Janus kinase inhibitors: A therapeutic strategy for cancer and autoimmune diseases. J Cell Physiol. (2020) 235:5903–24. doi: 10.1002/jcp.v235.9 32072644

[86] Wehde BL Rädler PD Shrestha H Johnson SJ Triplett AA Wagner KU. Janus kinase 1 plays a critical role in mammary cancer progression. Cell Rep. (2018) 25:2192–2207.e5. doi: 10.1016/j.celrep.2018.10.063 30463015 PMC6431084

