# Supplementary figures and images for: Pan-cancer single cell and spatial transcriptomics analysis deciphers the molecular landscapes of senescence related cancer-associated fibroblasts and reveals its predictive value in neuroblastoma via integrated multi-omics analysis and machine learning
Source: Front Immunol. 2024 Dec 5;15:1506256. doi: 10.3389/fimmu.2024.1506256 (PMC11655476; doi:10.3389/fimmu.2024.1506256)

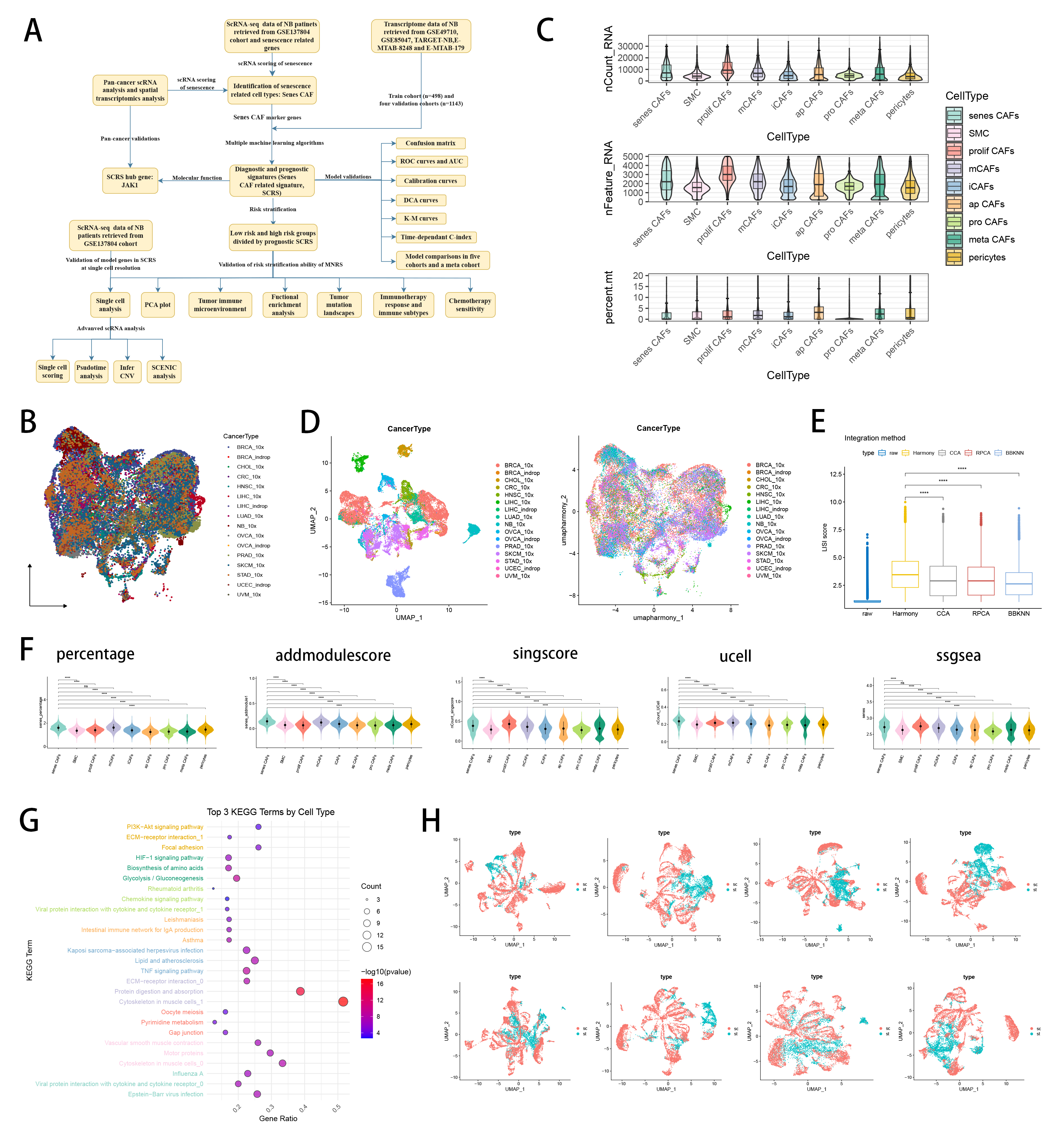

Supplement: Supplementary Figure 1 — (A) The workflow chart of our study. (B) Visualizing the distribution of various cancer types by UMAP plot in pan-cancer landscape. (C) Quality control of CAF subtype in pan-cancer scRNA-seq cohort. (D) Harmony algorithm reduced the batch effects between each cancer type. (E) Boxplot showing the local inverse Simpson’s Index (LISI) of fibroblasts before and after batch correction. (F) Five scRNA scoring algorithms visualized the senescence enriched scores of each CAF subpopulation. (G) KEGG enrichment analysis showed each CAF subtype’s top 3 functional terms. (H) UMAP plots showing the co-embedding results of scRNA-seq and ST data using CellTrek. [file Image1.jpeg]

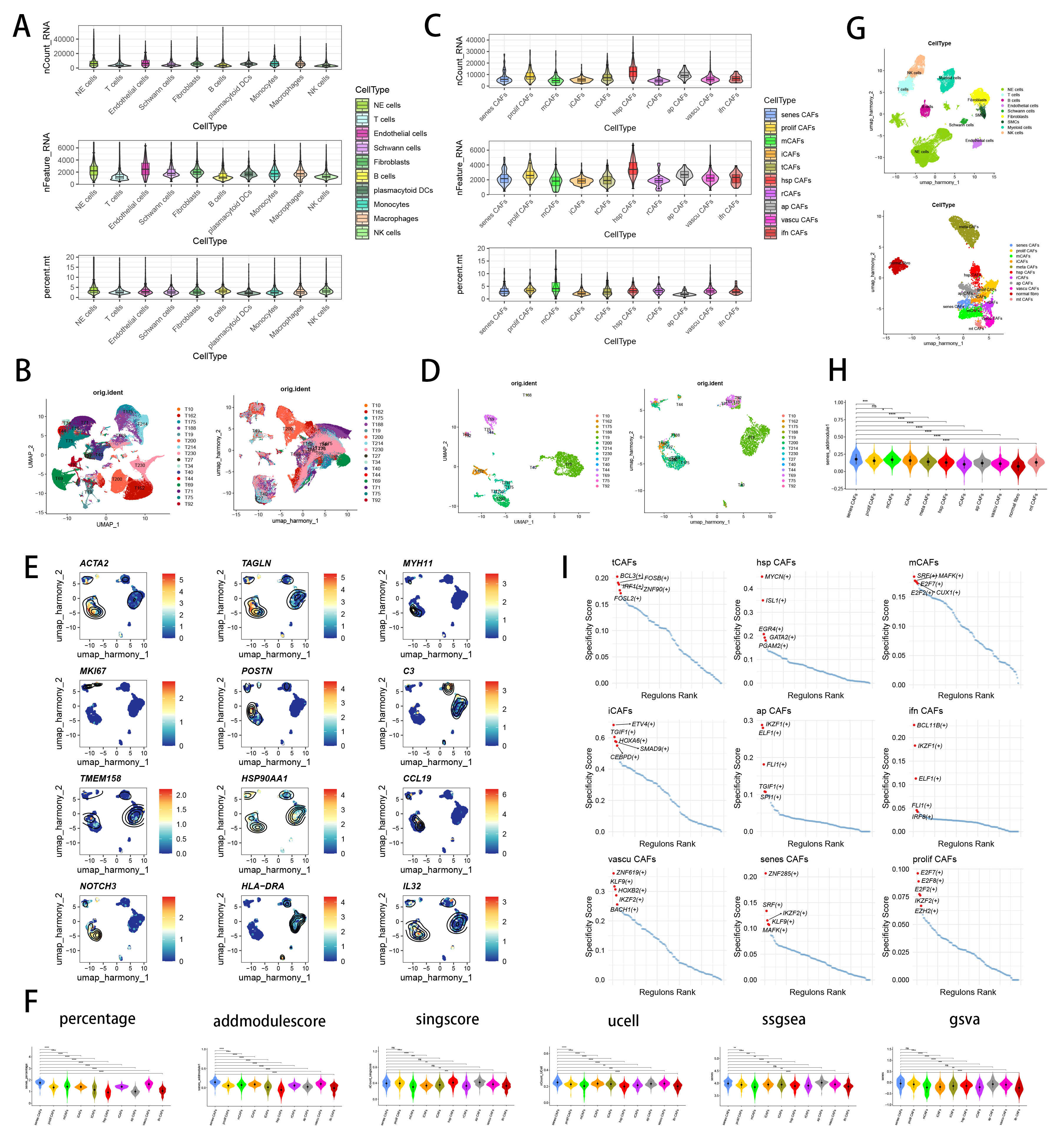

Supplement: Supplementary Figure 2 — (A) Quality control of each major cell type in GSE137804 scRNA-seq cohort. (B) Harmony algorithm reduced the batch effects of each sample in GSE137804 scRNA-seq cohort. (C) Quality control of each subtype in CAF subpopulations. (D) Harmony algorithm reduced the batch effects of each sample in CAF subpopulations. (E) UMAP plot showed the expression profiles of marker genes. (F) Six scRNA scoring algorithms visualized the senescence enriched scores of each CAF subpopulation. (G) Visualizing the distribution of major cell type and CAF subpopulations by UMAP plot in other NB datasets. (H) scRNA scoring algorithm of AUCell ranking visualized the senescence enriched scores of each CAF subpopulation. (I) Scatter plot showing the regulon specificity scores (RSSs) in each CAF subtype via SCENIC analysis. The top 5 regulons are highlighted. [file Image2.jpeg]

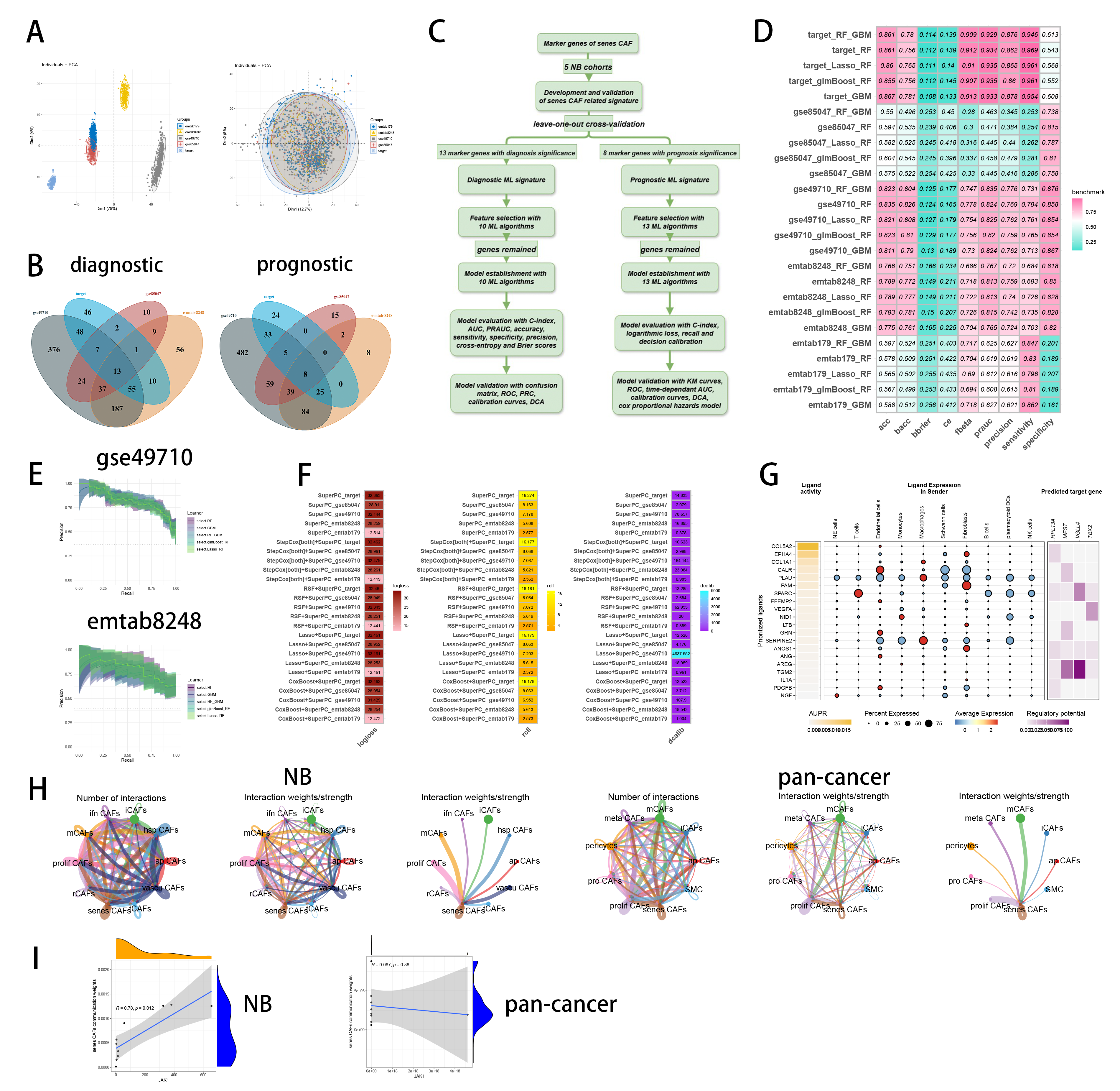

Supplement: Supplementary Figure 3 — (A) PCA plots visualized the well corrections after batch removement in five bulk-seq cohorts. (B) Acquisition of diagnostic and prognostic senescence-related genes via intersection of genes with diagnosis or prognosis value in four bulk-seq cohorts. (C) The flowchart to schematically explain the algorithmic pipeline of machine learning algorithm integration. (D) The performance of five ML models in terms of Area under precision-recall curve (PRAUC), accuracy, sensitivity, specificity, precision, cross-entropy, Brier scores, balanced accuracy and F1 Score in five bulk-seq cohorts. (E) The precision-recall curves (PRC) in two bulk-seq cohort. (F) Logarithmic loss, recall and decision calibration of top 5 prognostic machine learning models in five bulk-seq cohorts. (G) The combined heatmap shows the results after NicheNet analysis of high-SCRS cells and low-SCRS cells. (H) Circle diagrams showed the interaction strength and numbers between each CAF subtype in high-SCRS cells and low-SCRS cells in NB and pan-cancer landscape. (I) The spearman correlations between the hub gene of senes CAFs and cell-cell communication strengths in NB and pan-cancer landscape. [file Image3.tif]

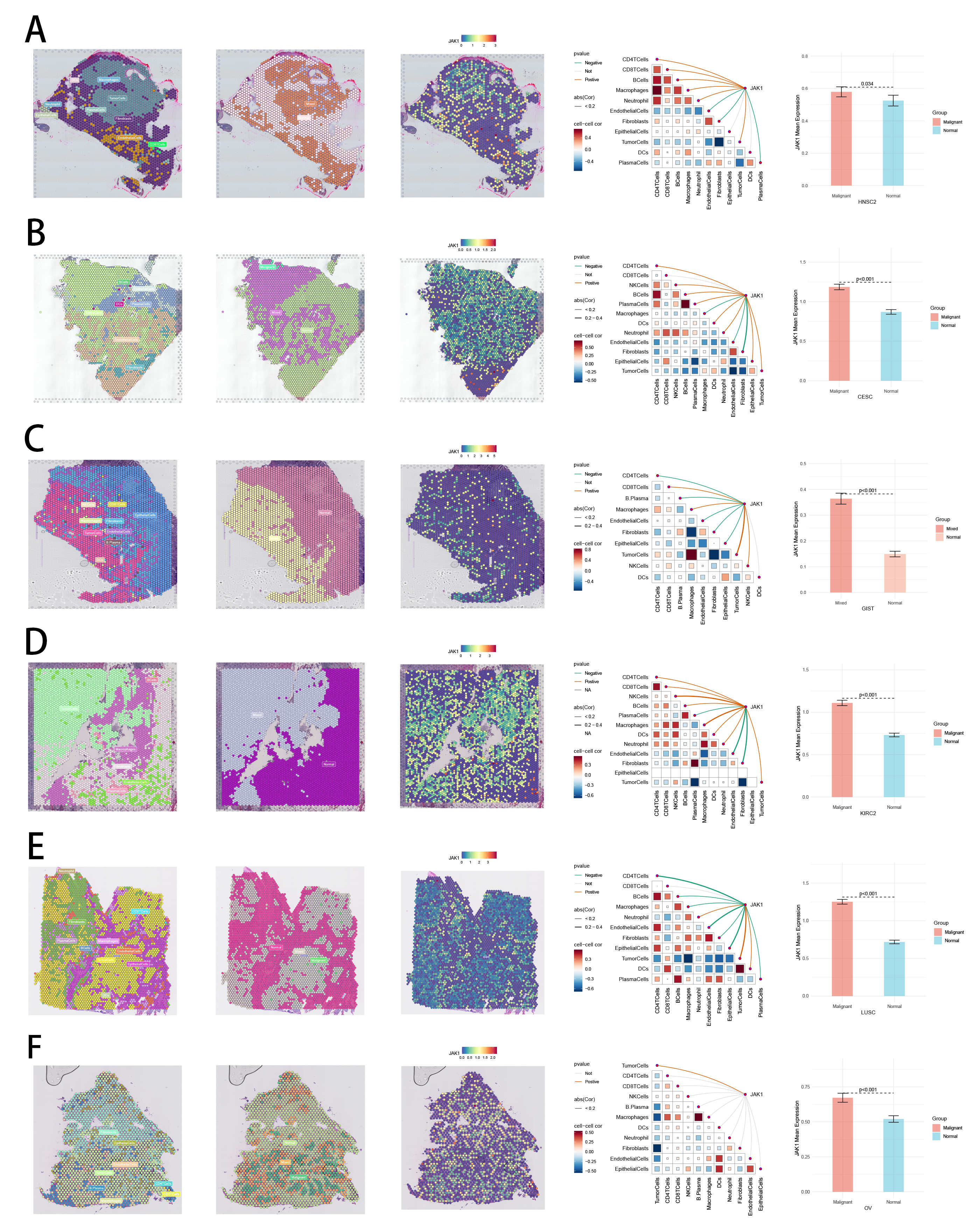

Supplement: Supplementary Figure 4 — Pan-cancer spatial transcriptomics analysis of JAK1 in HNSC (A), CESC (B), GIST (C), KIRC (D), LUSC (E) and OVCA (F). Left one: Each dot is a microregion of spatial transcriptome sequencing, and a different color represents a different cell type. Left two: Spatial feature plots of malignant, mixed and normal areas via “Cottrazm” analysis. Left three: Spatial feature plots of gene expression of JAK1. Left four: Spearman correlation analysis calculated the correlations between one cell count and another cell count, and between cell count and gene expression in all spots. Left five: The horizontal coordinate is the different microregion types, and the vertical coordinate is the average expression of JAK1. Wilcoxon Rank Sum Tests assessed the significance of statistical differences. [file Image4.jpeg]
